# Leveraging electronic health records and knowledge networks for Alzheimer’s disease prediction and sex-specific biological insights

**DOI:** 10.1038/s43587-024-00573-8

**Published:** 2024-02-21

**Authors:** Alice S. Tang, Katherine P. Rankin, Gabriel Cerono, Silvia Miramontes, Hunter Mills, Jacquelyn Roger, Billy Zeng, Charlotte Nelson, Karthik Soman, Sarah Woldemariam, Yaqiao Li, Albert Lee, Riley Bove, Maria Glymour, Nima Aghaeepour, Tomiko T. Oskotsky, Zachary Miller, Isabel E. Allen, Stephan J. Sanders, Sergio Baranzini, Marina Sirota

**Affiliations:** 1 Bakar Computational Health Sciences Institute, University of California, San Francisco, San Francisco, CA USA; 2 Graduate Program in Bioengineering, University of California, San Francisco and University of California, Berkeley, San Francisco and Berkeley, CA USA; 3 Memory and Aging Center, Department of Neurology, University of California, San Francisco, San Francisco, CA USA; 4 Weill Institute for Neuroscience. Department of Neurology, University of California, San Francisco, San Francisco, CA USA; 5 Department of Anesthesiology, Pain, and Perioperative Medicine, Stanford University, Palo Alto, CA USA; 6 Department of Pediatrics, Stanford University, Palo Alto, CA USA; 7 Department of Biomedical Data Science, Stanford University, Palo Alto, CA USA; 8 Department of Epidemiology and Biostatistics, University of California, San Francisco, San Francisco, CA USA; 9 Institute of Developmental and Regenerative Medicine, Department of Paediatrics, University of Oxford, Oxford, UK; 10 Department of Psychiatry and Behavioral Sciences, Weill Institute for Neurosciences, University of California, San Francisco, CA USA; 11 Department of Pediatrics, University of California, San Francisco, CA USA

**Keywords:** Alzheimer's disease, Machine learning, Ageing, Data integration, Predictive markers

## Abstract

Identification of Alzheimer’s disease (AD) onset risk can facilitate interventions before irreversible disease progression. We demonstrate that electronic health records from the University of California, San Francisco, followed by knowledge networks (for example, SPOKE) allow for (1) prediction of AD onset and (2) prioritization of biological hypotheses, and (3) contextualization of sex dimorphism. We trained random forest models and predicted AD onset on a cohort of 749 individuals with AD and 250,545 controls with a mean area under the receiver operating characteristic of 0.72 (7 years prior) to 0.81 (1 day prior). We further harnessed matched cohort models to identify conditions with predictive power before AD onset. Knowledge networks highlight shared genes between multiple top predictors and AD (for example, *APOE*, *ACTB*, *IL6* and *INS*). Genetic colocalization analysis supports AD association with hyperlipidemia at the *APOE* locus, as well as a stronger female AD association with osteoporosis at a locus near *MS4A6A*. We therefore show how clinical data can be utilized for early AD prediction and identification of personalized biological hypotheses.

## Abstract (web summary)

Identifying individuals at risk of developing Alzheimer’s disease is an important task. Here Tang et al. leverage electronic health records to predict Alzheimer’s disease onset, and utilize knowledge networks to prioritize shared genes behind the clinical data as well as facilitate contextualization based on sex.

## Main

Neurodegenerative disorders are devastating, heterogeneous and challenging to diagnose, and their burden in aging populations is expected to continue to grow^1^. Among these, AD is the most common form of dementia after age 65, and its hallmark memory loss and other cognitive symptoms are costly and onerous to both patients and caregivers. Approaches to curb this impact are moving increasingly to targeting interventions in at-risk individuals before the onset of irreversible decline^2–4^. To this end, advancements in AD biomarkers, diagnostic tests and neuroimaging have improved the detection and classification of AD, with approval of disease-modifying treatments, but there is still no cure and much remains unknown about its pathogenesis^5,6^. This is in part due to limited availability of longitudinal data or data linking molecular and clinical domains.

In the past few decades, electronic health records (EHRs) have become a source of rich longitudinal data that can be leveraged to understand and predict complex diseases, particularly AD. Prior applications of EHRs for studying AD include deep phenotyping of AD^7^, identification of AD-related associations and hypotheses^8^, and models classifying or predicting a dementia diagnosis from clinical data^9^. Data available in clinical records can also better represent a clinician’s knowledge of a patient’s clinical history at a point in time before further diagnostic studies or imaging, allowing a prediction model to be low cost to implement as a first-line application in primary care or for initial risk stratification^10^. While machine learning (ML) has been previously applied to EHRs for general dementia classification and prediction^11–14^, these approaches have limitations. These include limited specificity for the AD phenotype^15^, a lack of biological interpretability, imprecise temporal information or reliance on data modalities that may not be readily available in the EHR to facilitate early prediction (for example, neuroimaging^16–18^ or special biomarkers^19,20^). Sex as a biological variable is an important covariate for AD heterogeneity with potential contributions to differing risks and resilience, but sex-specific contributions have often been omitted from prior AD ML models^21,22^. Here we present an approach that utilizes vast EHR data for predicting future risk of AD with consideration of applicability and explainability of models.

With recent advances in informatics and curation of multi-omics knowledge, there is increasing interest in integrative approaches to derive insights into disease. Heterogeneous biological knowledge networks bring in the ability to synthesize decades of research and combine human understanding of multilevel biological relationships across genes, pathways, drugs and phenotypes, with vast potential for deriving biological meaning from clinical data^23^. There has been much AD research leveraging specific data modalities or combining a few modalities (transcriptomics^24,25^, genetics^26^ and neuroimaging^27^), but there is still a need for meaningful integration that allows for the understanding of the relationship between pathogenesis and clinical manifestations. Heterogeneous knowledge networks provide an opportunity to prioritize biological hypotheses from clinical data by synthesizing knowledge across multiple data modalities to explain relationships between many shared clinical associations^28,29^.

In this paper, we utilize EHR data from the University of California, San Francisco (UCSF) Medical Center to develop predictive models for AD onset and generate hypotheses of biological relationships between top predictors and AD. We carry out model construction and interpretation, controlling for demographics and visit-related confounding, to identify biologically relevant clinical predictors, and repeat with sex stratification. We demonstrate interpretability using heterogeneous knowledge networks (SPOKE knowledge graph)^30^ and validate predictors with supporting evidence in external EHR datasets and through genetic colocalization analysis. Our work not only has implications for determining clinical risk of AD based on EHRs, but also can lead to further research in identifying hypothesized early phenotypes and pathways to help further the field of neurodegeneration.

## Results

**Fig. 1:**
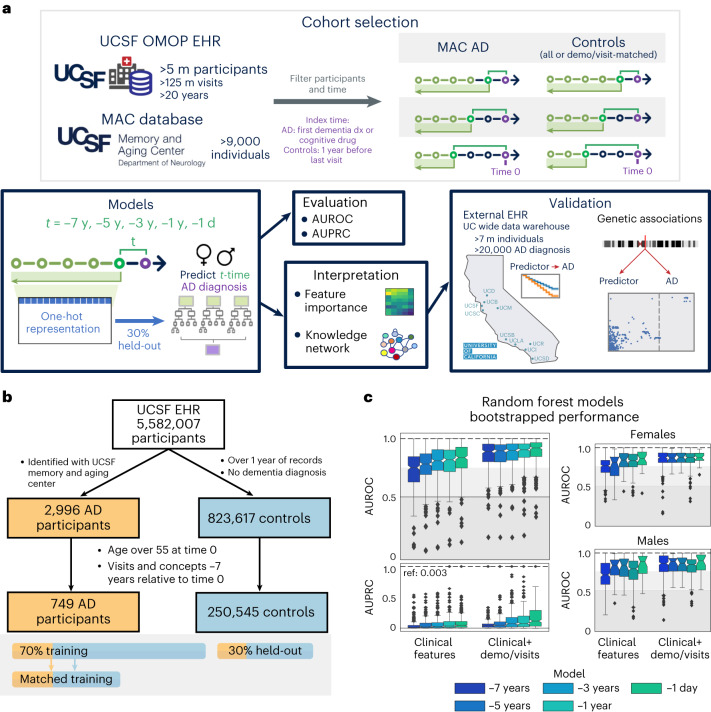
Overview of participant selection and RF model performance. **a**, From the UCSF EHRs and the UCSF Memory and Aging Center (MAC) database, participant and clinical information was extracted, filtered and prepared for time points before the index time. All clinical features extracted were one-hot encoded and trained on random forest (RF) models to predict future risk of AD diagnosis. Models were evaluated on a 30% held-out evaluation set to compute AUROC/AUPRC and interpreted based on feature importances and using a heterogeneous knowledge network (SPOKE). Top features were then further validated in external databases. **b**, Filtering a consistent set of individuals with AD and controls from the UCSF EHR for model training and testing. Filtered participant cohorts are shown in Table 1 and split with 30% held-out set for testing. **c**, Bootstrapped performance of RF models on the held-out evaluation set (*n* = 300 bootstrapped iterations of 1,000 participants, prevalence of AD on held-out set = 0.003). Bootstrapped AUROC performance for models trained and tested on female strata and male strata are also shown. The box shows quartiles (25th, 50th and 75th percentiles), whiskers extend to 1.5 times the interquartile range, and the remaining points are outliers.

**Extended Data Fig. 1:**
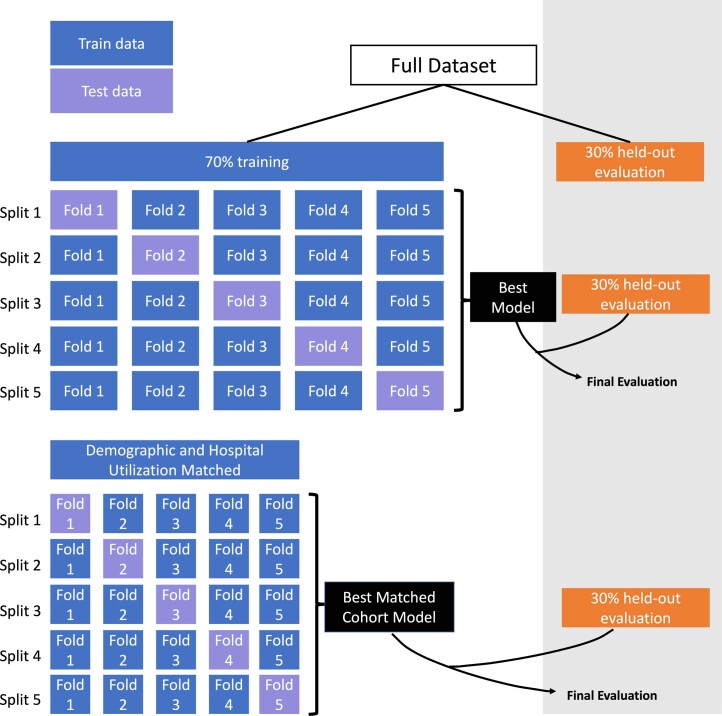
Cross-validation Approach. The full dataset was split into 70% for training and choosing the best model, and 30% was set aside as the held-out evaluation set. Model selection and optimization was performed with cross-validation on the 70% training set. All final models are then evaluated on the 30% held-out evaluation set.

From the UCSF EHR database of over 5 million people from 1980 to 2021, 2,996 individuals with AD who had undergone dementia evaluation at the Memory and Aging Center and thus had expert-level clinical diagnoses were identified and mapped to the UCSF Observational Medical Outcomes Partnership (OMOP) EHR database. From the remaining individuals, 823,671 controls were extracted with over a year of visits and no dementia diagnosis. After identifying an index time representing AD onset (mean onset age (s.d.), 74 (5.6) years; Methods) and filtering for availability of at least 7 years of longitudinal data, 749 individuals with AD and 250,545 controls were identified (demographics shown in Table 1). Of those, 30% were held out for model evaluation and 70% were utilized for model training (Fig. 1b and Extended Data Fig. 1). For each time point and within sex strata, ML models were either trained for AD onset prediction or trained on the AD cohort and a subset of propensity score-matched controls for hypothesis generation, where balancing was performed on demographics (sex, race and ethnicity, birth year, age) and visit-related factors (years in EHR, first EHR visit age, number of visits, number of EHR concepts and days since first EHR record; see example in Table 1 and Supplementary Table 4).

**Table 1:** Demographics of individuals used in models, and an example matched cohort for the −1-year model

All filtered individuals (pre-test/pre-train split)			
	Control	AD	
*n*	250,545	749	
**Age of AD onset (s.d.)**		74.0 (5.6)	
**Birth year, mean (s.d.)**	1945.5 (10.2)	1933.9 (5.3)	
**First visit age, mean (s.d.)**	51.2 (11.4)	57.0 (10.4)	
**Sex,** ***n*** **(%)**			
Female	139,548 (55.7)	468 (62.5)	
Male	110,829 (44.2)	281 (37.5)	
Nonbinary/unknown	168 (0.1)		
**R&E,** ***n*** **(%)**			
Asian/NHPI	32,427 (12.9)	151 (20.2)	
Black	17,111 (6.8)	62 (8.3)	
Latinx	15,036 (6.0)	53 (7.1)	
Other/unknown	28,177 (11.2)	45 (6.0)	
White	15,7794 (63.0)	438 (58.5)	
**Matched train individuals for −1-year model**			
	**Control**	**AD**	**SMD**
***n***	4,184	523	
**Birth year, mean (s.d.)**	1934.2 (5.6)	1934.0 (5.3)	−0.042
**First visit age, mean (s.d.)**	57.2 (9.4)	56.9 (10.5)	−0.028
**AD onset/index time age, mean (s.d.)**	74.1 (5.8)	74.1 (5.8)	−0.002
**Years in EHR, mean (s.d.)**	15.9 (7.8)	15.9 (7.9)	−0.004
**log(*n*** **prev visits), mean (s.d.)**	3.6 (1.5)	3.7 (1.6)	0.065
**log(*n*** **concepts), mean (s.d.)**	3.1 (1.3)	3.3 (1.4)	0.108
**log(days since first event), mean (s.d.)**	8.5 (0.4)	8.5 (0.4)	0.043
**Sex,** ***n*** **(%)**			0.094
Female	2,343 (56.0)	317 (60.6)	
Male	1,841 (44.0)	206 (39.4)	
**R&E,** ***n*** **(%)**			0.219
Asian/NHPI	705 (16.8)	112 (21.4)	
Black	520 (12.4)	35 (6.7)	
Latinx	280 (6.7)	39 (7.5)	
Other/unknown	223 (5.3)	32 (6.1)	
White	2,456 (58.7)	305 (58.3)	

The top half of the table shows characteristics of individuals in the UCSF EHR with visits and concepts over 7 years before index time. Care utilization information can be found in Supplementary Table 3. The bottom half of the table shows an example of training data where AD and controls are matched by the listed characteristics. Race & ethnicity (R&E) is a single variable derived from an algorithm developed by the UCSF Data Equity Taskforce^86^. log indicates natural logarithm. s.d. = standard deviation. NHPI = Native Hawaiian or Pacific Islander. SMD = standardized mean difference.

### ML models based on clinical data can accurately predict AD onset up to 7 years in advance

Random forest (RF) models trained on only clinical features from time points between 7 years and 1 day prior to AD onset were evaluated on the held-out dataset with average bootstrapped area under the receiver operating characteristic (AUROC) curve between 0.72 (median 0.75) for the −7-year time model and 0.81 (median 0.85) for the −1-day model. The RF models performed with area under the precision recall curve (AUPRC) greater than the reference held-out evaluation set AD prevalence of 0.003 (average/median of 0.05/0.01 for −7-year model and 0.10/0.06 for −1-day model, Fig. 1c). With addition of demographics and visit-related features, RF model performance improved with average bootstrapped AUROC between 0.86 (median 0.89) and 0.90 (median 0.94) and AUPRC between mean 0.06 (median 0.04) and 0.27 (median 0.14) for the −7-year and −1-day models, respectively (Fig. 1c).

**Extended Data Fig. 2:**
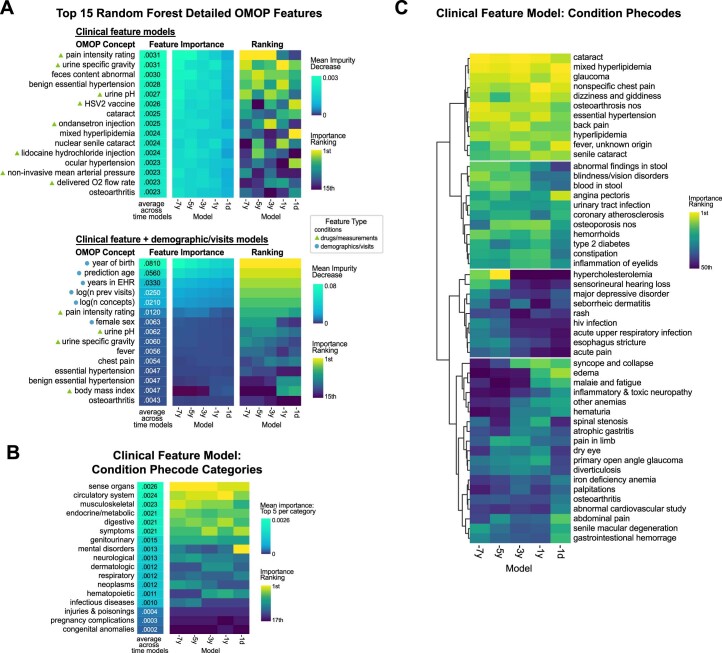
Top detailed features and phecodes from the random forest model. **a**. Top detailed OMOP clinical features utilized in models for clinical feature only models (top), or clinical features + demographic + visit information models (bottom). Features within the drug/measurement categories are marked with a triangle, while demographic/visit features are marked with a circle. **b**. Top phecode categories utilized in models, where importance is determined by the top 5 detailed features within each phecode mapping. The vertical order is based upon the average importance across time models. **c**. Top 50 phecodes utilized in time models, clustered based on relative importance across time models.

Top decision features across each time point model included features across clinical data domains, including vaccines, abnormal feces content, hypertension, hyperlipidemia (HLD) and cataracts (Extended Data Fig. 2a and Supplementary Data 1). Demographic and visit-related features became predictive for AD onset when added to the model (Extended Data Fig. 2a). EHR diagnoses mapped to phecode categories^31^ identified sense organs, circulatory and musculoskeletal phecode categories for early models, and mental disorder category for late models (Extended Data Fig. 2b). Among the top 50 ranked phecodes, one cluster identified phecode features that maintain high relative importance throughout the time models (HLD, hypertension, dizziness, abnormal stool contents), and other clusters contain features with relative importance at specific time points (Extended Data Fig. 2c). While some of these features support prior identified AD risk factors, the lack of adjustment may lead to feature identification as proxies for age in risk determination but not directly relevant to disease pathogenesis. Therefore, we proceed to identify disease-relevant features by training models on matched patients for the goal of hypothesis generation.

### Models trained on matched cohorts can identify hypotheses for biologically relevant AD predictors

**Extended Data Fig. 3:**
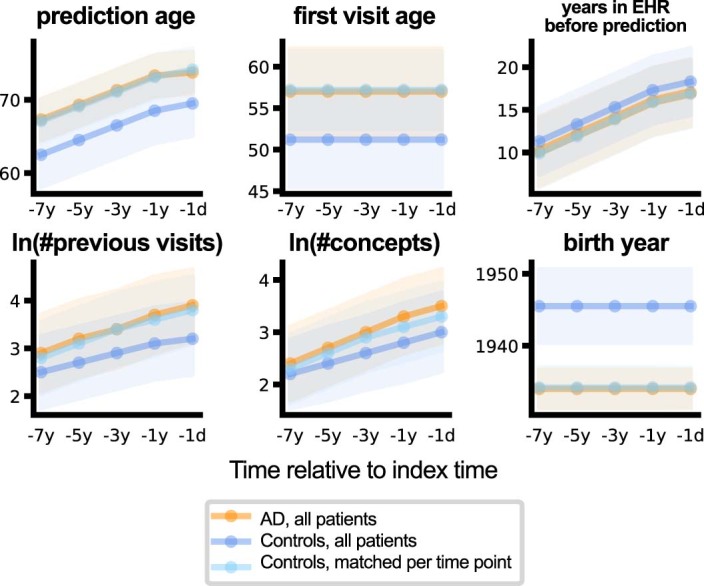
Comparison of age and visit-related factors between AD, controls, and matched controls. The plots demonstrate the distribution of continuous variables utilized in matching with error bands representing standard deviation. Orange represents AD patients at each time point. Dark blue represents all controls, while light blue represents controls that have been matched at each time point.

To train models that are robust for AD prediction for identifying predictors without demographic-related and visit-related confounding, we trained time point models on a matched set of participants at a 1:8 ratio between AD and controls. Sufficient balance was achieved on numerical covariates that were highly important in unmatched demographic models (Extended Data Fig. 3 and Supplementary Table 3).

**Fig. 2:**
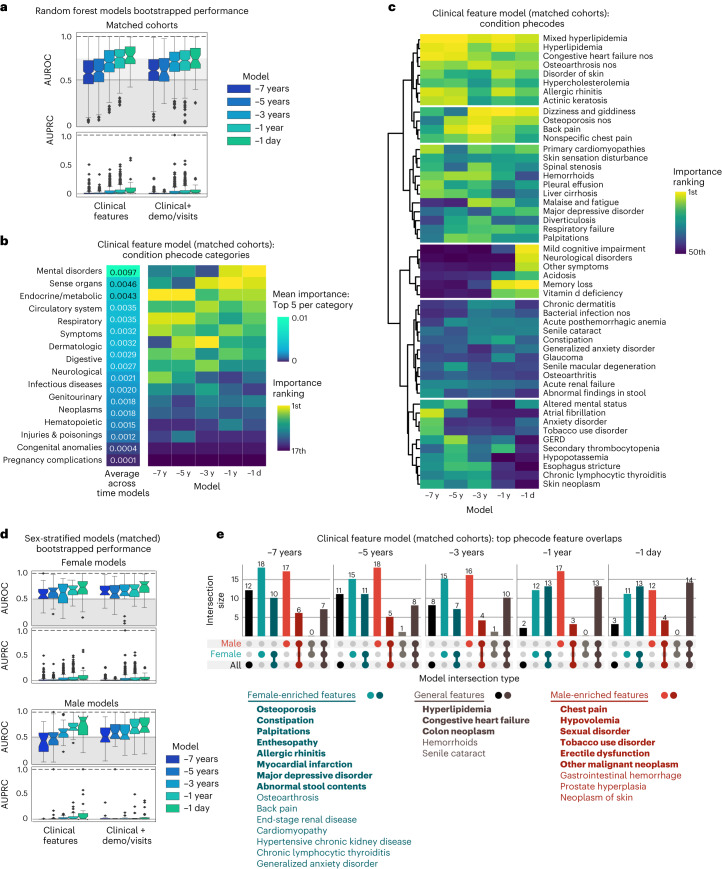
Models trained on matched cohorts allow for identification of hypotheses for AD predictors. **a**, Bootstrapped performance of models trained on cohorts matched by demographics and visit-related factors on the full held-out evaluation set (*n* = 300 bootstrapped iterations of 1,000 individuals, prevalence of AD on held-out set = 0.003). The box plot shows quartiles (25th, 50th and 75th percentiles), whiskers extend to 1.5 times the interquartile range, and the remaining points are outliers. **b**, Top clinical phecode categories for matched models ranked by the average of the top five importance values for each phecode category. Sorting is based on this average across time models. **c**, Top 50 phecodes (detailed features) across time models, with features clustered based on ward distance of rankings. **d**, Bootstrapped performances of sex-stratified matched models on the held-out evaluation set (*n* = 300 bootstrapped iterations of 1,000 individuals for each sex; reference AUPRC = 0.0036 female, 0.0022 male). Each box shows quartiles (25th, 50th and 75th percentiles), and whiskers extend to 1.5 times the interquartile range, with remaining points as outliers. **e**, Overlap of top matched model features for models trained on all individuals, female stratified individuals, and male stratified individuals, with model cutoff importance (RF average impurity decrease) greater than 1 × 10^−6^. Specific features are listed, with bold features indicating top features across all five time models and non-bolded features indicating top features across four time models.

RF models trained on only clinical features from −7 years to −1 day performed with average bootstrapped held-out evaluation set AUROC between 0.58 (median 0.57) for the −7-year model and 0.77 (median 0.77) for the −1-day model. The models performed with AUPRC greater than the held-out evaluation set AD prevalence of 0.003 with improvement closer to time 0 (mean/median of 0.02/0.008 for the −7-year model and 0.08/0.03 for the −1-day model; Fig. 2a). When demographics and visit-related information were added as features, the models performed with minimal to no improvement, with average bootstrapped test set AUROC between 0.61 (median 0.61) for the −7-year model and 0.71 (median 0.72) for the −1-day model and similar AUPRC (mean/median of 0.02/0.009 for the −7-year model and 0.05/0.03 for the −1-day model; Fig. 2a). For both the full and matched cohort models, the relative performances were consistent for balanced accuracy measures on the held-out evaluation, and a permutation test demonstrated significance for the −1-day matched cohort model (Extended Data Fig. 7).

Among top features sorted by average importance across models, top features include amnesia and cognitive concerns, HLD, dizziness, cataract, congestive heart failure, osteoarthritis and others (Fig. 2b). These top features are consistently important even when demographics and visit information were added to the model (Fig. 2b). Compared to models trained on all individuals, the models trained on matched cohorts have increased importance assigned to features like HLD and amnesia, while decreasing importance of features like pain intensity rating scale and essential hypertension (Extended Data Fig. 6).

Because matching allows for the control of the influence of visit-related and demographic-related information on AD prediction, the remaining diagnoses features can be identified for hypothesis generation with greater specificity for AD onset risk. Top phecode categories included mental disorders, sense organs and endocrine/metabolic categories (Fig. 2c). One cluster included features with maintained predictive importance throughout time models (HLD and congestive heart failure), while other clusters included phecodes that are relatively predictive several years before AD onset (osteoarthritis, allergic rhinitis). A cluster of features emerged as important around −3 years (osteoporosis, dizziness, back pain, hemorrhoids, palpitations) and some features only emerge as important closer to the time of AD onset (memory loss and vitamin D deficiency; Fig. 2c). Together, this shows that the model can identify a combination of conditions that can lead to AD risk identification for a patient of a given age and hospital utilization burden.

### Stratification by sex allows identification of features that are predictive within a subgroup

**Extended Data Fig. 4:**
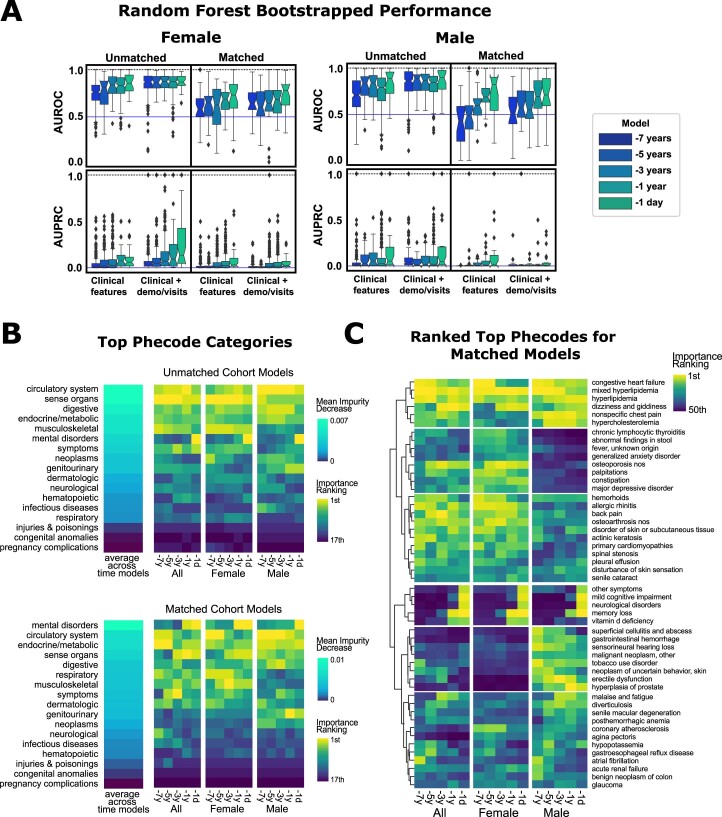
Sex stratified model performance and top features. **a**. The full performance of sex-stratified models is shown. The bootstrapped AUROC/AUPRC is determined by the male or female strata of the initial 30% held-out evaluation set (n = 300 bootstrapped iterations of 1000 patients for each sex, reference AUPRC = 0.0036 female, 0.0022 male). The box shows quartiles (25%, 50%, 75%ile), and whiskers extend to 1.5*interquartile range, with remaining points as outliers. **b**. Top phecode categories are listed by importance for all models, with inclusion of comparison with the general non-stratified model. Vertical ordering is determined by the average importance across time models. **c**. Top 50 important phecodes clustered by relative importance across time models and across strata.

Because sex plays a role in AD risk, models were trained within male-identified or female-identified sex groups to perform sex-specific prediction and identify sex-specific predictive features, without and with matching on demographics and hospital utilization (demographics in Supplementary Table 4). Models trained on clinical features performed with average held-out evaluation set AUROC between 0.75 (median 0.76) and 0.71 (median 0.71) for −7-year female and male models to 0.84 (median 0.86) and 0.82 (0.89) for −1-day female and male models. For AUPRC, the models performed greater than the held-out evaluation set prevalence (0.0036 for females, 0.0023 for males) with performance of 0.056 to 0.11 (median 0.022 to 0.061) and 0.041 to 0.15 (0.015 to 0.056) for female and male −7 year to −1-day time models, respectively. With addition of demographics and visit-related features, AUROC/AUPRC improved considerably (Extended Data Fig. 4a). Top features include sense organs and musculoskeletal phecode categories in female-only models, and circulatory system and digestive phecode categories as important among male-only models (Extended Data Fig. 4b).

To identify sex-specific biologically relevant clinical predictors for hypothesis generation, models were also trained by matching on demographic and visit-related factors within each subgroup (matching results in Supplementary Table 4). Time point models trained only on clinical features performed with mean held-out evaluation set AUROC of 0.60 to 0.68 (median 0.58 to 0.74) and 0.41 to 0.75 (median 0.43 to 0.84) for female and male models, respectively (Fig. 2d). For AUPRC, models performed greater than held-out evaluation set prevalence with performance ranging from 0.031 to 0.095 (median 0.0076 to 0.046) and 0.0040 to 0.125 (0.0033 to 0.022) for female and male models, respectively. Slight improvement in performance was observed with the addition of demographics and visit-related features (Fig. 2d).

Top phecode categories in the female models included respiratory/circulatory system features earlier on, to musculoskeletal features in the −5-year model, to sense organs and mental disorders in the later models. Top categories in male models included endocrine/metabolic/circulatory disorders earlier, to digestive and genitourinary disorders, to mental disorders in the −1-day model (Extended Data Fig. 4b). When comparing specific phecodes, some are general across the subgroups such as HLD, congestive heart failure (early models) and memory/cognitive symptoms (later models; Fig. 2e and Extended Data Fig. 4c). Female-driven features across time models included osteoporosis, palpitations, allergic rhinitis, myocardial infarction, major depressive disorder and abnormal stool contents. Male-driven features included chest pain, hypovolemia, sexual disorder, tobacco use disorder and neoplasms (Fig. 2e).

**Extended Data Fig. 5:**
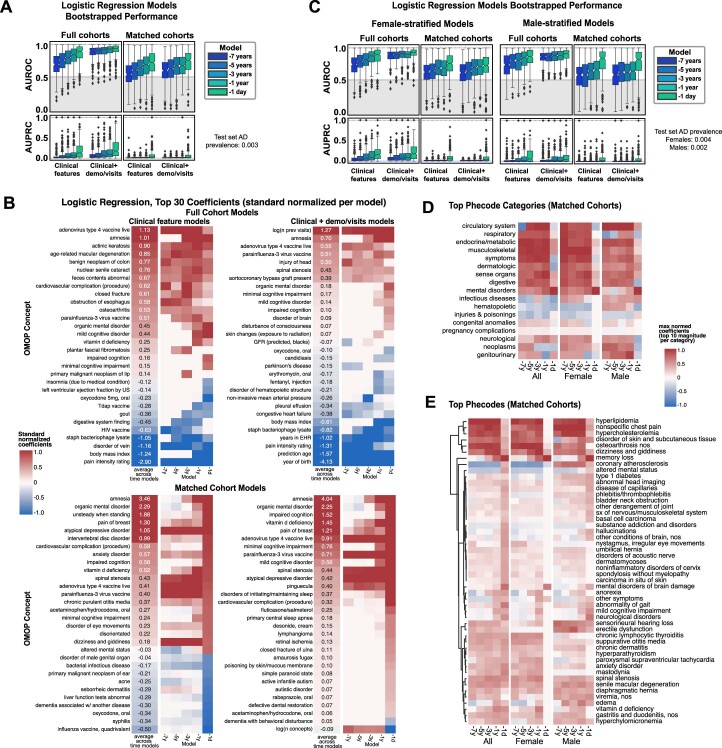
Logistic regression models and top coefficients. **a**. The full performance of logistic regression models. The bootstrapped AUROC/AUPRC is determined the 30% held-out evaluation set (n = 300 bootstrapped iterations of 1000 patients). The box shows quartiles (25%, 50%, 75%ile), and whiskers extend to 1.5*interquartile range, with remaining points as outliers. **b**. Top detailed OMOP feature logistic regression coefficients are listed by importance for all model formulations. Top row shows coefficients from the model trained on all patients, while the bottom row shows coefficients from the model trained on matched cohorts. **c**. The full performance of sex-stratified logistic regression models is shown. The bootstrapped AUROC/AUPRC is determined by the male or female strata of the initial 30% held-out evaluation set (n = 300 bootstrapped iterations of 1000 patients for each sex). The box shows quartiles (25%, 50%, 75%ile), and whiskers extend to 1.5*interquartile range, with remaining points as outliers. **d**. Top phecode categories across time models and across strata, determined by the top 10 logistic regression coefficient magnitudes within each category. **e**. Top 50 important phecodes clustered by average logistic regression coefficient across time models and across strata, where the average logistic regression coefficient is determined by the top 10 logistic regression coefficient magnitudes within each category.

**Extended Data Fig. 6:**
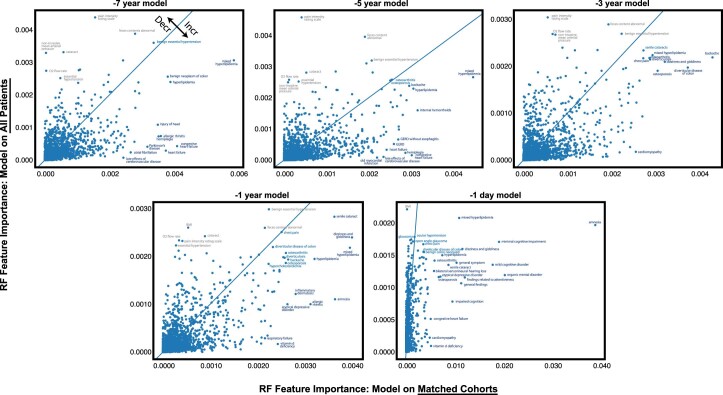
Random Forest Feature Importance Changes Between Models. A comparison of the random forest model feature importance between the model trained on all patients (y-axis) and the model trained on demographics/care utilization matched cohorts (x-axis). The blue line represents no change in feature importance. Above the blue line represents a decrease in feature importance in the model trained on the full cohort compared to matched cohorts, and below the line represents features with increased importance for the model trained on matched cohorts.

**Extended Data Fig. 7:**
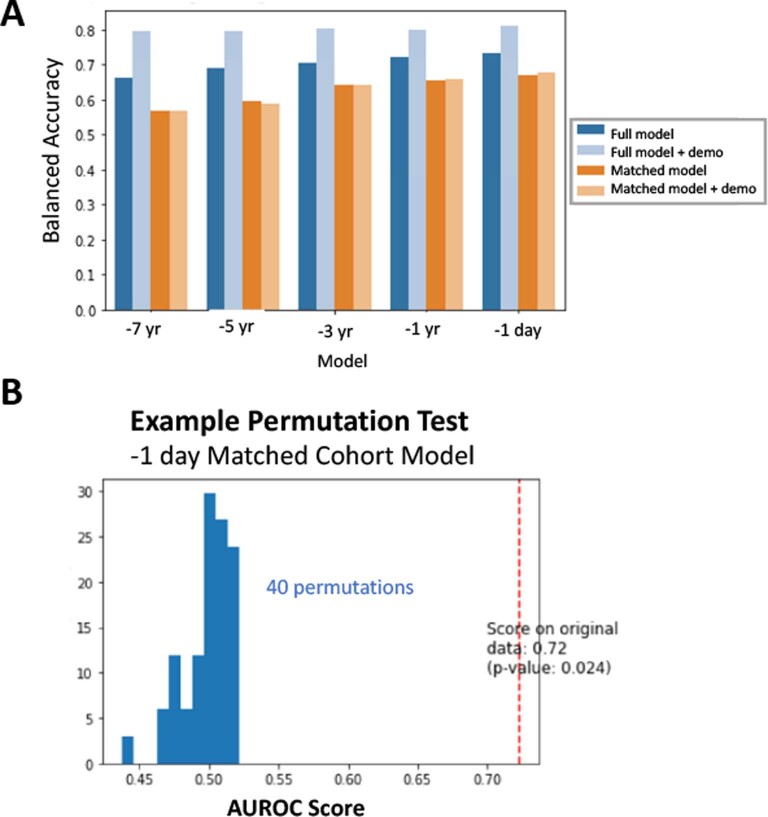
Balanced Accuracy and Example Permutation Test. **a**. Balanced accuracy on the 30% held-out evaluation set was computed for all random forest models. **b**. A null distribution for AUROC (score) was computed based on retrained random forest models with permutations on the ground truth label (40 permutations). P-value is calculated by (C + 1) / (n_permutations + 1), where C represents the number of permutations that scored better than the non-permuted dataset (see documentation for scikit-learn documentation of permutation_test_score function for associated paper and details).

For all formulations of the prediction task, logistic regression models performed comparably or worse to RF models and identified features with some overlap with those from RF models (Extended Data Fig. 5). For matched cohort models, RF performed better than logistic regression at the same time points (Supplementary Table 5) and identified decision features with nonlinear relationships with AD (for example, RF identified osteoporosis). Balanced accuracy measures for all the RF models supported similar trends in performance between models, including lower overall performance for matched cohort models, and improvement in model performance closer to AD onset (Extended Data Fig. 7a and Supplementary Table 6). As an example to evaluate the extent that clinical features meaningfully predict AD, RF models were retrained on permutations of the ground truth label (−1-day model, 40 permutations), and the trained model AUROC was significantly higher compared to the permutation distribution performance (*P* = 0.024, Extended Data Fig. 7b).

### Use of a knowledge graph allowed prioritization of potential biological explanations underlying predictive features

Next, we utilized the SPOKE knowledge graph^30^ to utilize existing knowledge to explain biological relationships between groups of top clinical model features and AD. We identified biological features (for example, genes, proteins and compounds) between the top 25 clinical predictors (mapped to disease nodes) and AD nodes for each model (Methods).

**Fig. 3:**
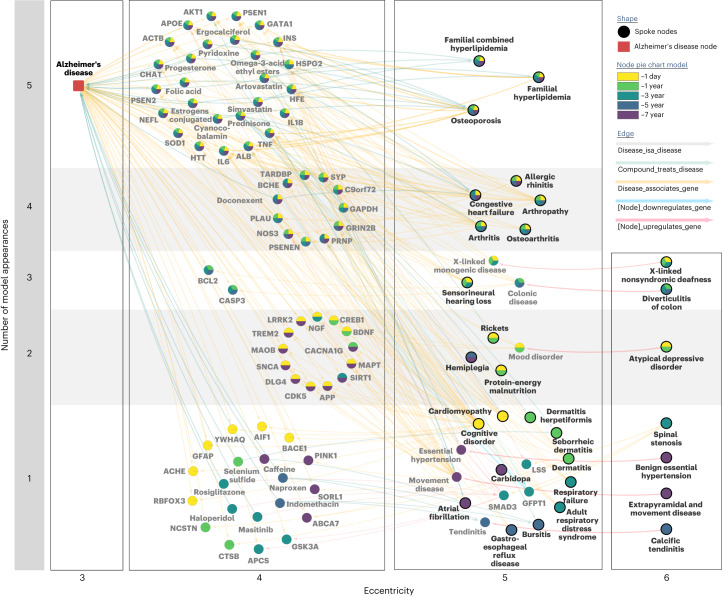
SPOKE provides biological prioritization of hypotheses associated with shared clinical phenotypes. Combined SPOKE network of all shortest paths to AD node (Disease Ontology ID: 10652) for the top 25 input features (bolded) from matched AD model at every time point. Network is organized based on the number of time point model occurrences (*y* axis) and eccentricity of a node in the subnetwork (*x* axis). Specific time point model occurrences are colored by the pie chart within each node.

Genes that appear in the shortest path networks among matched models across multiple time points included *APOE*, *AKT1*, *INS*, *ALB*, *IL1B*, *TNF*, *IL6* and *SOD1*, and compounds included atorvastatin, simvastatin, ergocalciferol, progesterone, estrogen, cyanocobalamin and folic acid (Fig. 3). These genes and compounds also shared relationships to multiple occurring model input nodes, particularly familial HLD and osteoporosis among all models across time (Fig. 3). Notable nodes that appeared over at least two models included C9orf72, *TREM2*, *APP* and *MAPT* with relationships to input nodes of musculoskeletal and joint disorders, deafness and depression (Fig. 3).

### Hyperlipidemia is validated as a top predictor of AD in external EHRs and a genetic link confirmed in *APOE* locus

**Fig. 4:**
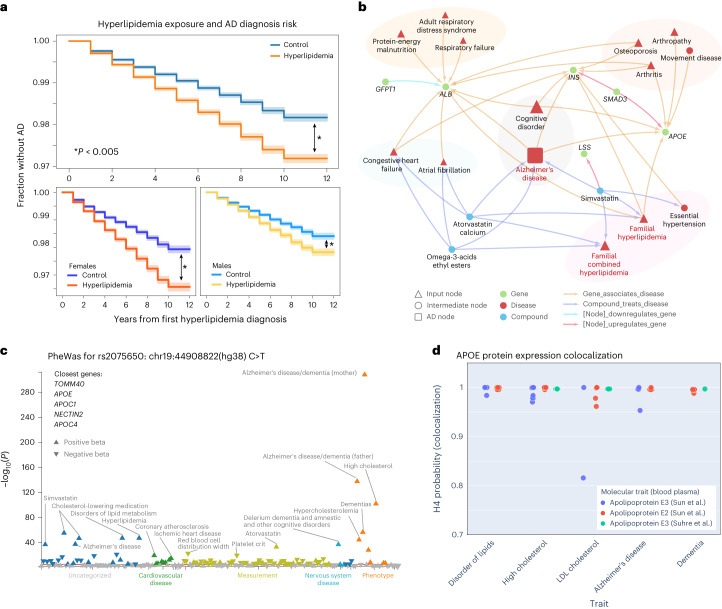
The HLD and AD association is validated externally with *APOE* as a shared causal genetic link. **a**, Kaplan–Meier curve on UC-wide EHR for HLD as the exposure (error bands show 95% CI). Two-sided log-rank test is significant for all HLD versus controls (*P* = 2.4 × 10^−85^), female HLD versus female controls (*P* = 3.6 × 10^−69^), and male HLD versus male controls (*P* = 8.4 × 10^−22^). *P < 0.005. **b**, First-degree and second-degree neighbors of HLD on the full network representing all shortest paths from the top 25 features per time model. **c**, PheWAS for variant rs2075650 (ch19:44892362(hg38):A > G) on a shared locus associated with both HLD and AD, plotted based on multiple prior studies with variant phenotype associations with *P* value < 0.05 from the UK Biobank. The red line indicates a Bonferroni-corrected significance level of 0.05 (191 phenotypes, Bonferroni *P* value = 0.00026), and the arrow direction represents the beta direction of effect of the alternative allele. **d**, Plot of APOE protein expression colocalization with H4 (probability two associated traits share a causal variant) from Open Targets Genetics. Each dot represents a specific phenotype categorized based on trait (*x* axis). Each color represents an APOE molecular trait measured from blood plasma from refs. ^100,101^.

**Extended Data Fig. 8:**
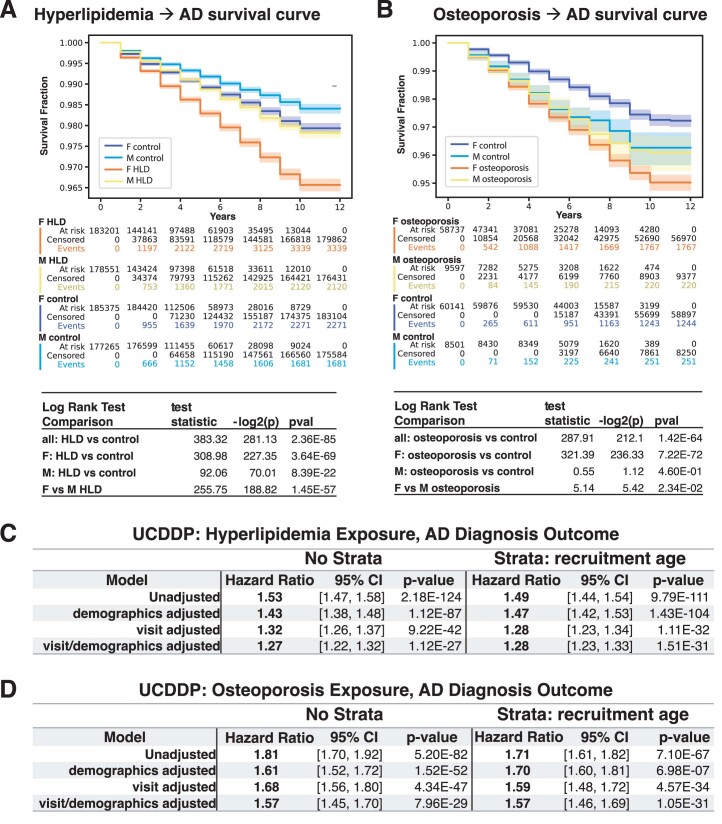
External EHR validation support increased AD diagnosis with hyperlipidemia and osteoporosis exposure. **a**. Sex-stratified combined Kaplan-Meier survival curves with hyperlipidemia (HLD) as the exposure (curve shows survival fraction, error bands show 95% confidence interval). Patient attrition is shown in the middle for each subgroup. Below, two-sided log rank test comparison results are shown. F = female, M = male. **b**. Sex-stratified combined Kaplan-Meier survival curves with osteoporosis as the exposure (curve shows survival fraction, error bands show 95% confidence interval). Patient attrition is shown in the middle for each subgroup. Two-sided log rank test comparison results are shown below. **c**. Hyperlipidemia exposure cox proportional hazard models for AD as the outcome, shown are the hazard ratios and 95% confidence intervals obtained from the exposure coefficient for unadjusted, demographic adjusted (gender, age, race, ethnicity), visit adjusted (first visit age, log(number of visits)), and demographic/visit adjusted. Right group shows computed hazard ratios with stratification by recruitment or starting age (age strata: <55, 55-60, 60-65, 65-70, 70-75, 75-80, >80). P-values are computed by a Wald’s test whose distribution is approximated by a Chi-squared test (two-sided) with one degree-of-freedom. **d**. Osteoporosis exposure cox proportional hazard models for AD as the outcome, shown are the hazard ratios and 95% confidence intervals obtained from the exposure coefficient for unadjusted, demographic adjusted, visit adjusted, and demographic/visit adjusted. Right group shows computed hazard ratios with stratification by recruitment or starting age (age strata: <60, 60-65, 65-70, 70-75, 75-80, >80). P-values are computed by a Wald’s test whose distribution is approximated by a Chi-squared test (two-sided) with one degree-of-freedom.

To further validate the utility of models to identify predictive disease associations, we followed up on hyperlipidemia (HLD) as a top feature that was a consistent predictor across all models. Utilizing a retrospective cohort study design in the EHR of five hospitals across the University of California system (University of California Data Discovery Platform (UCDDP)) with exclusion of UCSF, HLD-diagnosed individuals (exposed group, *n* = 364,289) had a faster progression to AD event compared to matched unexposed individuals (*n* = 364,289, matched demographics in Supplementary Table 7; Fig. 4a and Extended Data Fig. 8a, log-rank test *P* value < 0.005). This was further confirmed with a Cox proportional hazards analysis (hazard ratio (HR) 1.52 (95% confidence interval (CI) 1.46–1.57), visit/demographic-adjusted hazard ratio (aHR) 1.26 (1.21–1.31), *P* value < 0.005; Extended Data Fig. 8c).

To investigate potential relationships between HLD and AD, the HLD-specific knowledge network prioritized shared gene associations with *LSS*, *APOE*, *INS*, *SMAD3*, *ALB* and *GFPT1* (Fig. 4b). Locus intersections between high low-density lipoprotein (LDL) cholesterol and AD among two independent genome-wide association studies (GWAS) across 408,942 individuals with AD from ref. ^32^ and 94,595 individuals with high LDL cholesterol from ref. ^33^, respectively, identified multiple shared variants, including chr19:44892362(hg38):A > G (rs2075650) and chr19:44905579(hg38):T > G (rs405509). Phenome-wide association studies (PheWAS) for rs2075650 on the UK Biobank verified significant associations with cholesterol levels, HLD, AD and family history of AD (Fig. 4c). Colocalization H4 probability, a measure that determines the probability two traits are associated at a locus based on prior studies, supports a causal link with locus variants for APOE protein quantitative trait loci (QTL) and both HLD traits and AD traits (Fig. 4d).

### Female-specific predictor of osteoporosis is validated in an external EHR with potential explanations in SPOKE and genetic colocalization analysis

**Fig. 5:**
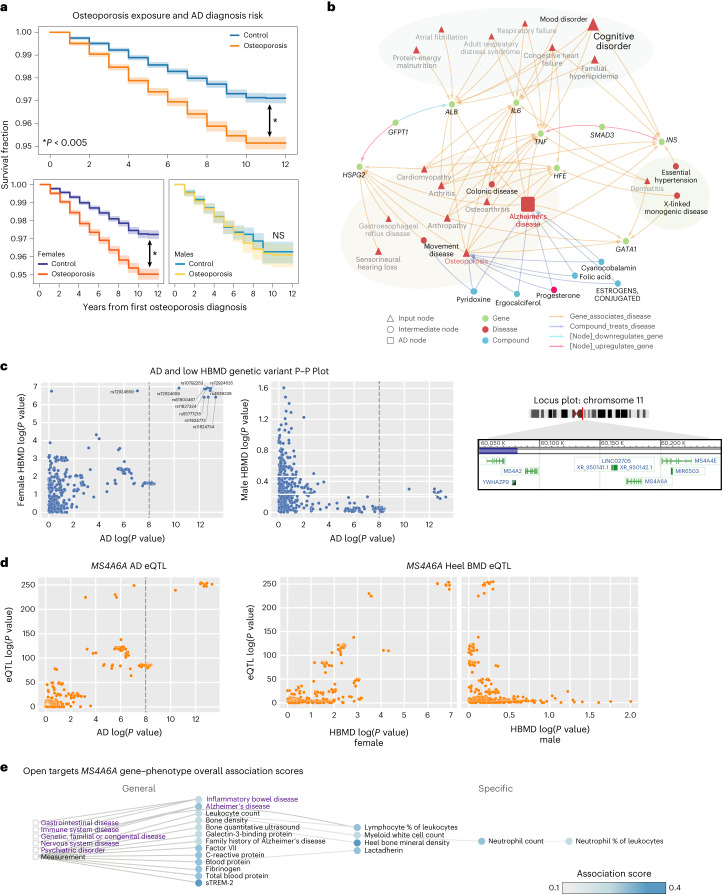
The association between osteoporosis and AD is validated externally with *MS4A6A* as a potential female-specific shared genetic link. **a**, Kaplan–Meier curve on UC-wide EHR for osteoporosis as the exposure (error bands show 95% CI). Two-sided log-rank test is significant for all osteoporosis-exposed individuals versus controls (*P* = 1.4 × 10^−64^) and osteoporosis-exposed female individuals versus controls (*P* = 7.2 × 10^−72^), but not male osteoporosis-exposed individuals versus controls (*P* = 0.46). *P < 0.005. **b**, First-degree and second-degree neighbors of osteoporosis node on the network representing all shortest paths from the top 25 features per time model. **c**, P–P plots between summary statistics of AD GWAS (*P* value computed as described in ref. ^35^, *n* = 455,258) and sex-stratified HBMD GWAS (female *n* = 111,152, male HBMD *n* = 166,988, *P* value computed as described in Neale’s Lab GWAS version 3) of variants around the *MS4A* locus (left and middle plots) at region 60050000–60200000 of chr11 (locus plot on right). **d**, *MS4A6A* gene expression (*cis*-eQTL, *P* values computed as described in ref. ^104^) association with AD GWAS (*P* value computed as described in ref. ^35^) and association with sex-stratified low HBMD (*P* value computed as described in Neale’s Lab GWAS version 3). **e**, Open Targets Genetics associated phenotype graph for *MS4A6A* with association score computed based on a weighted harmonic sum across evidence (described in https://platform-docs.opentargets.org/associations#association-scores/). Purple words indicate diseases, while black words indicate measurements. Circles are phenotypes colored by the association score, and boxes represent the most general categories. NS, not significant.

Osteoporosis was identified as an important feature in the matched models as a female-specific clinical predictor of AD. In the UCDDP, osteoporosis-exposed individuals (*n* = 68,940) showed a quicker progression to AD compared to matched unexposed individuals (*n* = 68,940, matched demographics in Supplementary Table 8; Fig. 5a and Extended Data Fig. 8b, two-sided log-rank test *P* value < 0.005). When stratified by sex, this progression was significant when comparing female individuals with osteoporosis (*n* = 57,486) versus female controls (*n* = 58,636, two-sided log-rank test *P* value < 0.005). Cox proportional hazards analysis further supported osteoporosis as a general risk feature for AD (HR 1.81 (95% CI 1.70–1.92), aHR 1.59 (1.45–1.70), *P* < 0.005; Extended Data Fig. 8d).

Osteoporosis-specific SPOKE network prioritized shared gene associations with *IL6*, *SMAD3*, *TNF*, *HSPG2*, *GATA1*, *GFPT1*, *HFE*, *INS* and *ALB* (Fig. 5b). Based on previous GWAS studies across 472,868 individuals with AD from ref. ^32^ and 426,824 participants with low heel bone mineral density (HBMD) from ref. ^34^, a shared risk locus was found in chromosome 11 between HBMD and AD among the *MS4A* gene family, with the closest gene as *MS4A6A*. A comparison of prior GWAS of up to 71,880 individuals with AD from ref. ^35^ and sex-stratified low HBMD GWAS (111,152 female, 166,988 male) of UK Biobank participants (https://www.nealelab.is/uk-biobank/) supports a female-specific association at the shared locus (Fig. 5c). Colocalization analysis supports a link between *MS4A6A* and AD (H4 = 0.987), female-specific HBMD with AD, and phenotypes with *MS4A6A* gene expression (Fig. 5d; AD versus female HBMD H4 = 0.998, *MS4A6A* gene expression versus female HBMD H4 = 0.997). This statistical significance was not replicated for male-specific HBMD GWAS (Fig. 5d; AD versus male HBMD H4 = 0.00263, *MS4A6A* gene expression versus male HBMD H4 = 0.00266). *MS4A6A* weighted associations with other phenotypes from the Open Targets Genetics platform found locus associations with many inflammatory phenotypes including C-reactive protein, lymphocyte percentage and neutrophil count (Fig. 5e).

## Discussion

While there is great potential for ML on clinical data, balancing clinical utility and biological interpretability can be challenging. To address this, we used thousands of EHR concepts to develop prediction models for expert-identified AD diagnosis and selected an index time suggesting AD onset. Cohort selection and data preprocessing is a crucial first step to identify available clinical measures and optimal ground truth that is close to biological AD and avoid overly optimistic model performance due to nonspecific AD or improper data preprocessing^36^. Our prediction model shows predictive power up to 7 years before the defined index time of AD onset with AUROC of 0.72 (and up to AUROC of 0.86 with additional demographic and care utilization features), which is comparable with other models in literature that utilize clinical data to predict less specific dementia or AD diagnosis^11,37^. An application of the full model includes determining early disease risk in primary care settings before time-consuming and costly detailed neuropsychological, biomarker or neuroimaging assessments (after which imaging or biomarker classification models can be utilized^13^). This can aid in identification of at-risk patients for follow-up or inclusion in early intervention or trials, with the 1-day prior model as suggesting possible AD onset to be considered at that visit to facilitate earlier AD diagnosis. Furthermore, interpretable models, such as RF models, can identify common decision point features and allow clinicians to understand what clinical features were used in determining prediction probability and assess the model output with greater trust compared to ‘black box’ models.

To identify early clinical predictors that may be biologically relevant for AD diagnosis, we trained models on individuals matched by pre-identified confounding variables such as demographics and visit-related features to account for their influence in AD prediction. ML models still retain the ability to predict AD diagnosis with mean AUROC over 0.70 after the −3-year model for RF models. Demographics and visit-related features minimally improved model performance, as matching increased the specificity of the task to predict AD onset controlled on demographics and visit-related features. In terms of clinical utility, the models trained on matched individuals provide predictive power for a given clinical scenario between two individuals with similar pre-test probability of AD risk (for example, same age and disease burden), with application of this model as a tool for determining post-test probability of future AD risk. Furthermore, by balancing on pre-identified confounders, top features may be interpreted with more biological relevance. For example, while we identified essential hypertension as an important feature in the models trained on the full cohort, this diagnosis became less important in the models trained on matched cohorts, suggesting hypertension may be nonspecific for AD and may instead be more directly related to aging or disease burden.

Our models trained on matched cohorts identify or strengthen known or suggested hypotheses for early predictors of AD, such as HLD as a feature for all time models. We also elucidate the relative importance of features years in advance, such as allergic rhinitis and atrial fibrillation as early predictors, osteoporosis and major depressive disorder as non-neurological predictors, and cognitive impairment and vitamin D deficiency as late predictors of AD. Some of these prior predictors, such as depression and vitamin D deficiency, have been previously implicated in AD risk^38–40^. These findings potentially support hypotheses suggesting AD can be associated with general aging or frailty, which might present in non-neurological body systems either before or concurrent with AD^41–45^. Furthermore, interpretation of these models allows for the identification of higher-order groups of predictors that may contribute to disease heterogeneity or together, contribute to AD risk. Nevertheless, while these models can identify hypotheses of predictive features, EHR data can still capture clinical biases or misdiagnoses, and further studies can investigate the influence of behavioral bias versus biological relevance.

We further trained models on sex-stratified subgroups (female versus male), with and without matching on demographics and visit-related covariates, to identify sex-specific clinical predictors. Given evidence that sex may influence different pathways to AD diagnosis^24,46,47^, it is important to consider how patient heterogeneity may impact the training, utility and interpretation of a prediction model. From the matched cohort models, we identified clinical features in each subgroup that were consistent with the general models, such as HLD as important in every model and memory loss as important in late models. Furthermore, we identified features that were sex specific, such as osteoporosis, major depressive disorder, allergic rhinitis and abnormal stool contents as predictors enriched among women, and chest pain, hypovolemia, prostate hyperplasia and sensorineural hearing loss as predictive among men. Further work can seek to disentangle the biological meaning of these sex-specific predictive features: whether they reflect sex-specific non-neurological manifestation of prodromal states, contributing risk factors or even sex biases in clinician evaluation and treatment (for example, bone density evaluation may arise more often after a fall). These models also demonstrate that for a heterogeneous disorder like AD, subgroup composition, like sex ratio of a cohort, can influence the performance and the features that are identified as important. Differences in subgroup size and AD prevalence may contribute to greater predictive performance among female strata models. AUPRC is particularly impacted by AD prevalence and can influence interpretation of the positive predictive value of models within each sex stratum. In terms of identified features, the higher preponderance of females leads to a sex-specific predictive factor, osteoporosis, being identified as a general predictive variable in the general group. This further indicates that both generalizable models and subgroup-specific models can provide valuable insight, both general and personalized, for a complex disease. Furthermore, in the context of ML fairness, the performance and identified features of general models may be influenced by the demographic make-up of the training population, just like how greater number and AD prevalence among females influence greater female-strata performance and identification of osteoporosis in our general models.

We utilized a heterogeneous knowledge network (SPOKE) to identify shared biological hypotheses underlying model-identified top clinical predictors and AD. By combining the shortest paths in SPOKE between top predictors and AD, we can prioritize nodes (for example, genes) that are consistently relevant for the higher-order combination of human data derived top clinical predictors and AD and give insight via prioritization and combination of relationships. First, we were able to identify known genetic associations with dementia based upon top diagnoses, such as through identification of known autosomal dominant early AD genes such as *APP* and *PSEN1/PSEN2* (ref. ^48^). Other genes identified with known associations with AD include *APOE*, *HFE* and *HSPG2* variants that impact AD risk^49–53^. An example of insight gained through SPOKE integration includes *ACTB* relating to AD^54,55^, sensorineural hearing loss^56^, arthropathy and arthritis^57^. The prediction model allows for the prioritization of *ACTB* for individuals with the common comorbidities of sensorineural hearing loss and arthropathy/arthritis with risk of AD (where the SPOKE informed connection linking sensorineural hearing loss, arthropathy, arthritis and AD all together through *ACTB* has not been previously implicated in literature).

The SPOKE network can also be leveraged to propose biological explanations based on common nodes and shared associations between clinical predictors identified from human data and AD. For example, *ALB* is identified through SPOKE as a shared genetic association between congestive heart failure, malnutrition, HLD and AD. While prior relationships have been identified between *ALB* and many individual diseases, each of those diseases also have many implicated genetic relationships. Leveraging human data through the predictive models allows for the prioritization of abundant gene connection with multiple disease predictors. Given gene *ALB* roles in pathways such as heme biosynthesis (Reactome R-HSA-189445), HDL remodeling (Reactome R-HSA-8964058) and insulin-growth like factor regulation (Reactome R-HSA-8964058), prioritization of mechanistic hypotheses linking *ALB*-related pathways with the pathophysiology of EHR-derived predictors (congestive heart failure, malnutrition, HLD) can be explored in future studies. Another example insight includes *INS* as a shared association between osteoporosis^58^, hypertension^59^, HLD^60^ and AD^61,62^. Prior studies have identified potential mechanisms underlying the relationship between energy utilization, lipid levels, nutrition and neurodegeneration (for example, Reactome R-HSA-1266738 and R-HSA-16368)^63–65^, and this analysis allows for prioritization of mechanistic hypotheses to be further explored. While these associations are included in the SPOKE network derived from evidence in the literature, the association of these genes with specific early clinical predictors is less established; thus, this analysis allowed us to identify a constellation of phenotypes and underlying genetic relationships observable in a clinical setting that, together, can lead a clinician to suspect future AD risk, prioritize molecular pathways for testing or personalized treatment, and guide biological hypotheses generation in AD pathogenesis for future studies.

To validate a few top clinical predictors, we utilized a hypothesis-driven approach to support the relationship between two identified features (HLD and osteoporosis) and progress to AD diagnosis in an external database across the University of California EHR system. For both phenotypes, the UC-wide EHR database supports a potential increased AD diagnosis risk due to evidence of decreased time to AD and increased hazard of AD diagnosis in patients exposed to the predictor of interest. The association between HLD and AD has been identified in prior clinical studies and systematic reviews^66–69^. In particular, *APOE* is a well-established associated genetic locus^70^, and *APOE* polymorphism is known to modify AD risk, particularly in individuals carrying the ε4 allele^71^. Many studies have also shown the association of *APOE* with elevated lipid levels and cardiovascular risk factors^72,73^. The validation of these well-known associations shows not only that our ML models on clinical data can pick up HLD as a risk factor, but also that by utilizing the SPOKE network, we can integrate known relationships in the literature to potentially explain the association between HLD and AD and identify the *APOE* locus as a potential shared causal mechanism as demonstrated in the colocalization results. Beyond the ability to identify known relationships, the SPOKE network also proposes biological explanations of higher-order shared associations between clinical predictors, such as *ALB* as a shared genetic association between congestive heart failure, malnutrition, HLD and AD, or *INS* as a shared association between osteoporosis, hypertension, HLD and AD. Prior studies have identified potential mechanisms underlying the relationship between energy utilization, lipid levels, nutrition and neurodegeneration^61,62,74^, although specific hypotheses of mechanistic relationships are an area for exploration in future studies.

The association between osteoporosis and AD is also validated to a lesser extent in clinical studies and meta-analysis^75,76^, with unclear but possible sex modification of this effect. Our study identifies osteoporosis as a predictor for AD among females before AD but shows less of a relative predictive effect for males compared to other clinical features. Nevertheless, it is still possible that shared relationships between osteoporosis and AD exist in males. A bone mineral density GWAS analysis of female patients shows a significant association with AD GWAS around the *MS4A* family locus, and this is further supported by *MS4A6A* eQTL colocalization with both AD and HBMD in females. These findings of osteoporosis as a potential sex-specific predictor of AD, with shared relationships through *MS4A6A*, is an unknown and unexpected result identified from single-hypothesis-driven follow-up from our prediction models. Prior studies have established the *MS4A* gene cluster as a risk for AD, with one study identifying the cluster based on Mendelian randomization^77^, and another that identified a stronger female-specific effect size for *MS4A6A*^78^. Some studies investigating the role of the *MS4A* family suggest mechanisms that involve immune function, particularly among microglia^79^. While this gene may not have been identified in SPOKE, SPOKE did capture direct pathways through known genes involved in inflammation such as *IL6* and *TNF*, and we also observed *MS4A6A* as being highly associated with measurements of immune cells in the blood. Further studies will be needed to validate the exact associative mechanism between osteoporosis and AD, although some prior hypotheses suggest the potential impact of genetic variants on osteoclast function, amyloid clearance or oxidative stress response^80,81^. While we utilized knowledge networks to leverage knowledge to explain relationships between groups of predictors, we performed hypothesis-driven analysis on independent EHRs and genetics to further explore and validate a few chosen predictors (HLD and osteoporosis) with AD. Hypothesis-driven approaches can be applied to any other selected predictor or phenotype identified by the models to understand their relationships with AD onset that may not yet be represented by the knowledge graphs.

This study has several limitations. First, EHR data complexity and quality can affect prediction models, and it is challenging to distinguish the influence of clinician/patient behavior, sociological factors or underlying biology on identification of features. Matching can improve interpretability by removing the influence of non-biological covariates, but follow-up validation of hypotheses across omics data types is needed. Due to changing patient demographics and societal factors, prediction models should be continuously trained, updated and evaluated if implemented in the clinical setting to ensure effective utilization and account for biases that may have been learned from the data. Model utilization should investigate the impact of cohort selection biases and matching methods on model generalizability, and model retraining and calibration should be a continual aspect of model application to account for possible data drifts and changing clinical practice approaches that would arise in the future. Second, clinical EHR data are sometimes sparse and provide a superficial interval snapshot of a patient’s health, so the absence of a record may not necessarily reflect the absence of a condition and prior health information may not be available in the EHR. Therefore, the EHR provides a representation of an interval of a patient’s health history and is more likely to pick up diagnosis of chronic or common conditions, as well as common drugs or measurements. Future work can investigate the impact of variations in data representation that can account for data sparsity, continuous laboratory result outcomes, and best temporal assignment of diagnosis onset beyond binary representation or considering drug prescriptions for assignment of diagnoses. Third, survival models have extensive right censorship and do not consider competing risks. Fourth, because AD is heterogeneous and differential diagnosis is nuanced and subjective even in expert hands, predictive performance can be limited by label quality and the signal from clinical features can be noisy, limiting performance and generalizability. Future work investigating heterogeneity may identify subgroup-specific features where subgroups can be divided based on biotype, dementia syndromes, racialization, and so on. Future applications with hierarchical models, transfer learning or fine-tuning on a subpopulation can increase personalization of models. Fifth, our sex-stratified analysis was restricted to individuals who identified as female or male. Future studies could explore AD patterns among intersex individuals. Lastly, predictive features identified are relevant before AD onset, and future work is needed to identify diagnostic-relevant AD comorbidities, or conditions that can occur after AD progression. Because predictive features are identified as hypotheses, the direct mechanism and causal pathway relating a phenotype to AD are unknown. Future work can investigate causality with Mendelian randomization or mechanistic studies.

In this study, we demonstrate how formulation of prediction models can influence utility for predictive application or biological interpretation. We show how models can be used to identify early predictors, and utilize SPOKE to explain relationships via shared biological associations. Lastly, we show that our models can pick up known associations with HLD through *APOE*, and identify a lesser-known association with osteoporosis through *MS4A6A* that may be female specific. This study contributes to the field of EHR integrative research that can inform future directions in both AD care and research.

## Methods

### Ethical approval

This study complies with all relevant ethical regulations and is approved by the Institutional Review Board of UCSF (IRB 20-32422).

### Participant identification

Individuals with AD were identified based on UCSF Memory and Aging Center database containing over 9,000 participants mapped to the UCSF OMOP-format EHR. These individuals have undergone dementia evaluation at the Memory and Aging Center and thus had expert-level clinical diagnoses. In clinical settings, since AD is often a syndromic diagnosis indicating general dementia for memory or cognitive concerns^82–84^, we aimed to identify a highly accurate cohort diagnosed by neurodegeneration specialists to obtain an AD diagnosis that is closer to the biological ground truth^85^. The remaining control participants were obtained from the rest of the UCSF EHR, with over 1 year of records and no existing records of dementia diagnosis among the G[123]* International Classification of Diseases 10th Revision (ICD-10) categories (Supplementary Table 1). These controls include patients seen at the UCSF Memory and Aging Center with EHR data, but without a dementia diagnosis given.

To best build models for prediction of AD onset, an index time was determined to identify input model features before first clinical indication of dementia. This was defined among the AD cohort as the first time of any AD diagnosis, dementia diagnosis or prescription of cognitive drug (ATC codes N06D; Supplementary Table 2) to be the first time point of possible biological AD manifestation. This approach was utilized because individuals with AD may be prescribed an anticholinesterase inhibitor or given an alternative dementia diagnosis before a formal confirmation of an AD diagnosis. For controls, the index time was defined as 1 year before the last recorded visit date, with no dementia diagnosis given within that year. To maintain a consistent patient population for training and evaluation of ML models, the final AD and control cohort was identified by including participants who are at least 55 years of age at the index time and have existing clinical visits and concepts 7 years before the index time. These participants were then split into 70% for model training and tuning, while the remaining 30% were held-out for model evaluation (Extended Data Fig. 1). For sex stratification, we utilized sex as reported in the UCSF EHR (male, female), excluding nonbinary and other categories due to low sample size, as a proxy for representing sex as a biological variable.

### Data extraction and preparation

Demographics (birth year, gender, race and ethnicity), clinical concepts (conditions, drug exposures, abnormal measures) and visit-related features (age at prediction, first visit age, years in UCSF EHR) were extracted before the index time for the AD and control cohort from the UCSF OMOP EHR database. Race and ethnicity is a single variable derived from an algorithm developed by the UCSF Data Equity Taskforce to codify aggregated sociopolitical categorizations based on EHR self-reported identifiers^86^. To train models in advance of the index time, clinical information was extracted for each participant including all clinical data up to a time point *X* before the index time, where *X* includes −7 years, −5 years, −3 years, −1 year and −1 day. These time points represent the knowledge of a participant’s clinical history leading up to time *X* before time. All existing clinical features (conditions, drug exposures, abnormal measurements) were one-hot encoded. Abnormal measures were extracted from the OMOP measurement table based on the numeric value falling either above range_high or below range_low, and abnormal measures were binary encoded based on abnormal flagging, following the approach from ref. ^29^. If a clinical feature did not exist or if the clinical measure was within normal range, the encoding is represented as a 0 and therefore assumed to be normal. As the UCSF database only captures an interval of a participant’s interaction with the healthcare system, prior non-chronic conditions may not be captured within the EHR.

Demographic and visit-related features (prediction age, first visit age, years in UCSF EHR, log(number prior visits), log(number prior concepts), log(days since first clinical event)) were scaled between 0 and 1 on the training data, where log indicates natural logarithm and feature scaling allows for multiple ML model approaches. Age at prediction is defined at the age of the participant at which the model is applied (for example, if a participant index time is at age 70, then the age of prediction for the −5-year model is 65). All features with no variance were removed for each model, with the total number of features ranging from 5,211 features (−7-year model on matched cohorts) to 23,760 features (−1-day models on unmatched cohorts). Information about input features, specific OMOP concepts and select top feature prevalences can be found in Supplementary Data 1.

### ML preparation and training

Binary classification time point models for AD were trained using the participant representation at each time point before the index time. We divided the data into two sets—70% for model creation and 30% for evaluation. Training and optimal model selection (with hyperparameter tuning) was performed on the 70% split with cross-validation, and 30% was held out for evaluation and not seen during model training and selection in any way. Final selected model evaluation was performed on the 30% held-out evaluation set as the common dataset to obtain and compare the performance of all final models (diagram in Supplementary Fig. 1). Models were trained with clinical features only (clinical model) and with clinical features plus demographics and visit-related information (clinical plus demographics/visits model). Models were also trained on samples matched by demographics and hospital utilization to account for biases and confounding in prediction. In these models, control participants were matched to individuals with AD at a 1:8 ratio on demographics (birth year, race and ethnicity, sex) and visit-related features (age, first visit age, years in EHR, log(number of prior visits), log(number of prior concepts), log(days since first clinical event)) utilizing propensity score matching^87^ (propensity score estimated based upon a logistic regression model, nearest-neighbor matching without replacement). While propensity score is often utilized to balance treatment probabilities in cohort studies, it has also been utilized for sample selection^88,89^, exposure likelihood^90^ or for outcome-based case–control studies^7,91^.

RF models were primarily utilized for both predictive performance and interpretability that take into account the high collinearity between clinical variables. RF models were trained using the scikit-learn package^92^, with balanced class weight parameter. Hyper-parameters were tuned (grid search) based on cross-validation performance (5-fold) of AUROC on the 70% model training set to determine parameters of *n*_estimators (*n*_features, *n*_features × 2, *n*_features × 3), max_depth (3, 5, 7, 9) and max_features (sqrt, log_2_). The number of estimators and maximum depth were tuned to balance between performance and overfitting, while a subset of features (max_features) was utilized per tree to help account for high correlation between features^93,94^. Models were evaluated on bootstrapped subsamples (300 iterations, 1,000 samples) of the 30% held-out evaluation set to determine AUROC and AUPRC for model comparability. Balanced accuracy scores were also computed on the 30% held-out evaluation set. An elastic net logistic regression model was also trained on both the full and matched cohorts for comparison. We performed a permutation test on the −1-day matched cohort model to determine the significance of AUROC compared to a null distribution of AUROC scores of models trained from permuted ground truth labels (40 permutations) to determine the extent to which clinical features can be predictive of AD.

#### Stratification

Both models for full participant cohorts and matched cohorts were re-performed in sex strata using the same method based upon sex reported in the UCSF EHR to augment the OMOP database. Models were trained on two sex subgroups—female and male—due to lack of other subgroups labeled in the EHR. For each stratum, individuals with AD were re-matched to controls within each stratum for the matched participant trained models. Models were evaluated similarly based on AUROC/AUPRC on the same bootstrapped held-out evaluation set, stratified by sex.

### Top feature interpretation

RF models were investigated for feature interpretation due to the combined interpretable nature of the models (compared to neural networks) and the ability to capture nonlinear relationships (compared to logistic regression models)^95^. Average gini impurity decrease for each feature was utilized to evaluate the importance of each feature in the RF models (feature importance). The average importance for each feature was taken across each time point model (−7 years, −5 years, −3 years, −1 year and −1 day) to obtain an across-model importance for each model type, and normalized by the maximum importance value across all time point models within each model type (for example, RF) and group (for example, female strata). Feature importances were then ranked within each model to obtain relative importance within each of the time points.

As a patient’s exposure to a medication or a laboratory test is often a result of a diagnosis, we pursued interpretability based on diagnostic features that have been mapped to phecodes, which is a semi-manual hierarchical aggregation of meaningful EHR phenotypes^31^. This allows for a lossy categorization of detailed OMOP features (OMOP IDs) to phecodes (OMOP ID → SNOMED → ICD-10 → phecode) and phecode category. SNOMED IDs were mapped to ICD-10 based upon recommended rule-based mappings from the National Library of Medicine September 2022 release (https://www.nlm.nih.gov/healthit/snomedct/us_edition.html). ICD-10 codes were then mapped to phecodes based on the release from ref. ^96^. To obtain the importance within each phecode or phecode category, the average importance for the top five detailed OMOP features per phecode or phecode category was computed, and ranked between phecodes or categories. For phecodes across all models and sex-stratified models, the ranking of importance of phecodes across each time model was hierarchically clustered with Ward linkage.

To compare top phecodes between sex-stratified models to identify sex-specific features, top RF features over an average importance threshold of 1 × 10^−6^ were identified per time model trained on matched participants. Upset plots were then generated for each time point based upon this overlap. Female-driven features were defined as features that exist in both the full model and female models, or only female models, and male-driven features were defined analogously.

### UC-wide validation analysis with hypothesis-driven retrospective cohort analysis

Two top clinical features were selected from the matched all-participant model (HLD) and matched sex-specific models (osteoporosis) and further followed up on an external EHR database to validate the feature as predictive and conferring risk for AD diagnosis. With these features defined as exposures, hypothesis-driven analysis was performed with a retrospective cohort study design on the University of California hospital EHR database (UCDDP) with exclusion of any patients seen at UCSF, so with included institutions consisting of UC Davis, UC Los Angeles, UC Riverside, UC San Diego and UC Irvine. Exposed participants were identified with the exposure (HLD or osteoporosis), which were identified by string-matching and mapping to all descendants or related concepts based on the OMOP relationship tables, and final SNOMED codes are shown in Supplementary Tables 6 and 7. Controls were identified among the remaining participants. Recruitment age was defined as the age of exposure diagnosis (for exposed cohort) or the first visit age in the visit_occurrence table (for unexposed or control cohort), which was then matched to represent the start of the cohort study timeline. All participants were then filtered to have at least 2 years of records in the EHR, and last visit age was utilized for right censorship.

The outcome of interest was AD diagnosis, which was identified based on SNOMED codes 26929004, 416780008 and 416975007 (Supplementary Table 5). Exposed and control (unexposed) groups were then matched based on demographics (gender, race and ethnicity), birth year and recruitment age (propensity score estimated based upon a logistic regression model, nearest-neighbor matching without replacement). We utilized the gender_id column to identify sex, as the standard documentation intend for this column to represent biological sex (https://ohdsi.org/web/wiki/doku.php?id=documentation:vocabulary:gender/). Note that only two options exist (female concept_id = 8532 and male concept_id = 8507), and that accurate sex and gender information may be limited depending on the institution or EHR collection of sex information.

Analysis of time to AD diagnosis includes utilization of Kaplan–Meier survival curves fitted with 95% CIs and two-sided log-rank test to compare survival curves between groups. Sex-stratified curves were also fitted. Cox proportional hazard models were utilized to obtain unadjusted HRs and aHRs by demographics and/or visit information, with and without stratification by recruitment age or birth year, and with 95% CIs.

### Heterogeneous network analysis

Heterogeneous knowledge networks, such as SPOKE, integrate known relationships across biological and phenotypic data realms in databases and literature. Such a network could provide hypotheses to explain relationships between groups of phenotypes that may not be immediately known^23,28^. We proceeded with interpretation on the matched models, with the top 25 model features taken for each time point and mapped to SPOKE nodes based on ref. ^29^. Note that mappings may not be 1 to 1. All shortest paths were then computed from each input node to the AD node (DOID: 10652), and the shortest paths were filtered to exclude certain node types (Anatomy, SideEffect, AnatomyCellType,Nutrient) and edges (CONTRAINDICATES_CcD, CAUSES_CcSE, LOCALIZES_DlA, ISA_AiA, PARTOF_ApA, RESEMBLES_DrD). Edges were also filtered based on the following criteria: TREATS_CtD at least phase 3 clinical trial, UPREGULATES_KGuG/ DOWNREGULATES_KGdG *P* value at most 1 × 10^−4^, PRESENTS DpS enrichment at least 5 and Fisher *P* value at most 1 × 10^−4^.

If multiple detailed OMOP features map to the same node, the importance of the node was obtained by the average of OMOP feature importances. Networks for all time models were combined into a single network (union of nodes and edges), and total node importance was determined by the maximum across time. Network metrics were then computed with the Cytoscape ‘Network Analyzer’ function^97^. The combined time model networks were then sorted by eccentricity metric on the *x* axis (representing maximum distance to all other nodes, with the lower number representing higher importance) and the number of individual time model network occurrences in the *y* axis (showing node importance persistence across time). With this layout, highly traversed nodes in the shortest paths between multiple EHR informed top model features and AD can be identified and prioritized for hypothesis generation and further investigation. Note that due to the heterogeneous nature of edges and lack of edge weighting, distances in the figures are not meaningful.

To focus on two selected features for the full matched model (HLD) and the female-specific matched model (osteoporosis), the combined network was filtered based on first-degree and second-degree neighbors of the starting feature of interest. This allows for visualization of associated genes and AD, as well as relationships with other top model features found from the clinical models.

### Validation with genetic datasets

We further explored the association between clinical predictors and AD by identifying shared genetic loci between top model phenotypes and AD, based on colocalization probability and weighted evidence association scores computed from Open Targets Genetics^98,99^ (https://genetics.opentargets.org/). Colocalization analysis is a method that determines if two independent signals at a locus share a causal variant, which helps increase the evidence that the two traits (for example, HLD and AD, or protein expression and AD) also share a causal mechanism. It is a Bayesian method which, for two traits, integrates evidence over all variants at a single locus to evaluate the following hypothesis that two associated traits share a causal variant. This is the H4 probability.

We first identified shared loci between the selected phenotypes (HLD or osteoporosis) and AD by identifying the genetic intersection between AD and related phenotypes in Open Targets Genetics.

For HLD and AD, we utilized the Open Targets Genetics platform to identify overlapping variants and shared loci between LDL cholesterol and family history of AD or phenotype AD (https://genetics.opentargets.org/study-comparison/GCST002222?studyIds=GCST90012878/). PheWAS between a shared genetic variant and UK Biobank phenotypes were plotted and extracted from the Open Targets Genetics platform. Colocalization analysis tables between the gene, molecular RNA or protein expression, and phenotypes were extracted, with apolipoprotein E protein QTL for *APOE* gene specifically identified based on blood plasma quantity data from refs. ^100,101^.

Similarly for osteoporosis and AD, we utilized the Open Genetics platform to identify shared loci between HBMD (proxy for osteoporosis) and family history of AD or phenotype AD (https://genetics.opentargets.org/study-comparison/GCST006979?studyIds=GCST90012877/). To further investigate the shared locus, we extracted GWAS summary statistics from ref. ^35^ for AD and sex-stratified GWAS summary statistics for low HBMD from Neale’s Lab GWAS round 2, phenotype code: 3148, based on data from the UK Biobank (www.nealelab.is/uk-biobank/)^102^. We then conducted colocalization analysis using the coloc method described in ref. ^103^, from R package coloc 5.1.0. Summary statistics for *MS4A6A* *cis*-eQTL in blood were extracted from eQTLGen^104^, and colocalization analysis was performed between AD, sex-stratified low HBMD and *MS4A6A* eQTL on the locus region 60050000–60200000 of chromosome 11 (locus image from NCBI Genome Data Viewer). To investigate further associations with the locus, *MS4A6A* associations with all other phenotypes were extracted from Open Targets Genetics platform with inclusion of weighted literature evidence association scores.

### Statistics and reproducibility

All analyses were performed on datasets where data collection was completed previously. While randomization is not possible in observational datasets like the EHR, we used propensity score matching, an approach in causal inference to match by probability of group membership, to enable identification of matching case and control groups and mimic randomization. Quality of matching can be assessed with standardized mean difference of relevant covariates. Blinding is not applicable to this study. Inclusion and exclusion of participants are described in the above sections and summarized in Fig. 1b to ensure specificity of groups and observed time frames. No further data were excluded from analyses.

No statistical method was utilized to predetermine sample size. For all statistical analysis, non-parametric tests were used if normality is not assumed about the data distribution, otherwise normal distribution was assumed but not formally tested.

### Reporting summary

Further information on research design is available in the Nature Portfolio Reporting Summary linked to this article.

### Supplementary information

Supplementary Information Legends for Supplementary Tables 1–4 and Supplementary Tables 5–9

Reporting Summary

Supplementary Data 1 Excel file with information regarding model inputs, model importances, top condition prevalences and raw data for molecular validation. The ‘model input’ tabs lists the number of input features per model (OMOP concepts or other), and a description of demographic or visit-related features. The ‘top model importance’ tab lists the top 50 trained random forest model importance values, full and matched, and sex-stratified models, with the mapped phecodes. The ‘top feature prevalences’ tab shows the prevalence of some of the top conditions utilized in prediction. ‘SexModelTopFeatOverlap’ shows top features over importance threshold of 1 × 10^−6^ for the sex-specific models across time, and extent of overlap between models relevant for the upset plot. The sheets starting with ‘rf’ list all model inputs and associated OMOP concept_ids (m: matched cohorts utilized, dv: demographic and visit-related features added, −7/−5/−3/−1/0: years before AD onset for prediction with 0 representing the −1-day model). The ‘rs2075650_PheWAS’ tab shows original *P* value and source of study for the PheWAS figure associated with the variant. The ‘APOE_pQTL_coloc’ tab shows Open Target colocalization query results for *APOE* with phenotype traits and blood protein expression.

Supplementary Table 1 List of mappings from ICD-10 codes G[123]* to OMOP codes for determining exclusion of controls. The mapping was generated and manually reviewed to create a white-list of certain codes and approve exclusion of dementia-related codes.

Supplementary Table 2 List of mappings from dementia/frontotemporal disorder-related condition concepts to SNOMED OMOP mappings and N06D ATC code to RxNorm OMOP mappings for identifying index time 0 for individuals with AD.

Supplementary Table 3 Demographics of matched cohorts (propensity score matched by demographics and visit-related factors; Methods) on the training set for matched cohort models.

Supplementary Table 4 Demographics of male and female cohorts (combined train and test set). The same patients for train/test set split in the general model are utilized for the sex-stratified models. Matched cohorts on the sex-strata training sets are also shown for the sex-specific matched cohort models.

## Data Availability

EHR concepts and identification approaches are described in the Methods, and concepts are derived from the OMOP common data model structure (Supplementary Tables 1 and 2). Model inputs and importances can be found in Supplementary Data 1. Phecodes can be downloaded at https://phewascatalog.org/phecodes_icd10 or https://phewascatalog.org/phecodes, and mappings between ICD-10 codes and SNOMED can be accessed at https://www.nlm.nih.gov/healthit/snomedct/us_edition.html. Data for UK Biobank phenotype GWAS summary statistics can be found at https://www.nealelab.is/uk-biobank/, and eQTL data can be downloaded from https://www.eqtlgen.org/. For the P–P and eQTL plots, documentation for the Open Targets API can be found at https://www.genetics.opentargets.org/api/. Access to EHR databases and participant-identifiable information are controlled due to the sensitive nature of the data. The UCSF EHR database can be accessed by UCSF-affiliated individuals by contacting UCSF Clinical and Translational Science Institute (ctsi@ucsf.edu) or UCSF’s Information Commons team (info.commons@ucsf.edu). If the reader is unaffiliated with UCSF, they can set up an official collaboration with a UCSF-affiliated investigator by contacting the principal investigator, M.S. Participant data from the UCSF Memory and Aging Center can be requested at https://memory.ucsf.edu/research-trials/professional/open-science/ or through a collaboration with a principal investigator affiliated with the UCSF Memory and Aging Center. Requests should be processed within a couple of weeks. UCDDP is only available to UC researchers who have completed analyses in their respective UC first and have provided justification for scaling their analyses across UC health centers (more details at https://www.ucop.edu/uc-health/departments/center-for-data-driven-insights-and-innovations-cdi2.html or by contacting healthdata@ucop.edu). The SPOKE knowledge network can be accessed at https://spoke.rbvi.ucsf.edu/neighborhood.html. More details about the network can be found in ref. ^30^ and mappings to EHR concepts can be found in ref. ^29^.

## Code availability

Code for prediction models can be found at https://github.com/al1563/ADprediction_code/. Other code can be made available upon request. Relevant packages include Python scikit-learn version 0.23.2, scipy version 1.2.0, joblib version 1.1.0, lifelines version 0.27.4, tableone version 0.7.12 and R coloc version 5.1.0.
