# Supplementary material for: Leveraging electronic health records and knowledge networks for Alzheimer’s disease prediction and sex-specific biological insights
Source: Nat Aging. 2024 Feb 21;4(3):379–95. doi: 10.1038/s43587-024-00573-8 (PMC10950787; doi:10.1038/s43587-024-00573-8)
Supplement: Supplementary file 1 — Legends for Supplementary Tables 1–4 and Supplementary Tables 5–9 [file 43587_2024_573_MOESM1_ESM.pdf]

nature aging

Analysis

https://doi.org/10.1038/s43587-024-00573-8

Leveraging electronic health records and knowledge networks for Alzheimer's disease prediction and sex-specific biological insights

In the format provided by the authors and unedited

Supplemental Table 1: Control exclusion codes.

List of mappings from ICD-10 codes G[123]* to OMOP codes for determining exclusion of Controls. The mapping was generated and manually reviewed to white-list certain codes and approve exclusion of dementia-related codes.

Supplemental Table 2: Dementia codes.

List of mappings from Dementia/FTD related condition concepts to SNOMED OMOP mappings and N06D ATC code to RxNorm OMOP mappings for identifying index time 0 for AD patients.

Supplemental Table 3: Matching results for time point models on matched cohorts

Demographics of matched cohorts (propensity-score matched by demographics and visit-related factors, see Methods) on the training set for matched cohort models.

Supplemental Table 4: Male and female demographics and matching result

Demographics of male and female cohorts (combined train and test set). The same patients for train/test set split in the general model are utilized for the sex-stratified models. Matched cohorts on the sex-strata training sets are also shown for the sex-specific matched cohort models.

Supplemental Table 5: Matched cohort trained model comparison between logistic regression and random forest

Mean and standard deviations AUROC was computed for bootstrapped samples of the held-out evaluation set for both the random forest and logistic regression models for comparability.

Model Time	Bootstrapped mean AUROC	Bootstrapped std AUROC	Model Type	Features
-1 day	0.771667	0.139762	random forest	clinical
-1 yr	0.738797	0.16183	random forest	clinical
-1 day	0.70313	0.20803	logistic regression	clinical
-3 yr	0.695912	0.183331	random forest	clinical
-1 yr	0.674981	0.189248	logistic regression	clinical
-3 yr	0.637145	0.187386	logistic regression	clinical
-5 yr	0.598022	0.207104	random forest	clinical
-5 yr	0.589743	0.197771	logistic regression	clinical
-7 yr	0.583837	0.206874	random forest	clinical
-7 yr	0.549061	0.192763	logistic regression	clinical

Model Time	Bootstrapped mean AUROC	Bootstrapped std AUROC	Model Type	Features
-1 day	0.738133	0.170242	random forest	clinical + demo/visits
-1 day	0.728514	0.182411	logistic regression	clinical + demo/visits
-1 yr	0.710432	0.183543	random forest	clinical + demo/visits
-3 yr	0.700636	0.175239	random forest	clinical + demo/visits
-1 yr	0.663312	0.187115	logistic regression	clinical + demo/visits
-3 yr	0.657146	0.198652	logistic regression	clinical + demo/visits
-5 yr	0.619819	0.201044	logistic regression	clinical + demo/visits
-7 yr	0.60619	0.181737	random forest	clinical + demo/visits
-5 yr	0.599069	0.199292	random forest	clinical + demo/visits
-7 yr	0.59507	0.199938	logistic regression	clinical + demo/visits

Supplemental Table 6: Balanced accuracy performance of models
Balanced accuracy (defined as average recall for both the positive and negative class) performance on the held-out evaluation set for both the full model and the matched cohort trained model.

Model Time	Full or matched cohort model	Held-out evaluation set balanced accuracy	Features
-7 yr	full	0.66333567	clinical
-5 yr	full	0.68713887	clinical
-3 yr	full	0.70504803	clinical
-1 yr	full	0.72020014	clinical
-1 day	full	0.73342356	clinical
-7 yr	matched	0.56751959	clinical
-5 yr	matched	0.59464602	clinical
-3 yr	matched	0.64351437	clinical
-1 yr	matched	0.65277962	clinical
-1 day	matched	0.67079665	clinical

Model Time	Full or matched cohort model	Held-out evaluation set balanced accuracy	Features
-7 yr	full	0.79420735	clinical + demo/visits
-5 yr	full	0.79553055	clinical + demo/visits
-3 yr	full	0.80227966	clinical + demo/visits
-1 yr	full	0.79940679	clinical + demo/visits
-1 day	full	0.81155760	clinical + demo/visits
-7 yr	matched	0.56836172	clinical + demo/visits
-5 yr	matched	0.58730455	clinical + demo/visits
-3 yr	matched	0.64186021	clinical + demo/visits
-1 yr	matched	0.65705140	clinical + demo/visits
-1 day	matched	0.67644997	clinical + demo/visits

Supplemental Table 7: UCDDP AD patient concepts and demographics

Top table shows the specific concepts utilized to identify Alzheimer's Disease as the outcome in the UCDDP database, with breakdown by number of patients per concept. Due to deidentification, only a patient's birth year is known for age estimation.

**Term**	**# patients**
Alzheimer's disease	20562
Primary degenerative dementia of the Alzheimer type, senile onset	9327
Primary degenerative dementia of the Alzheimer type, presenile onset	2530

		**Overall**
**n**		24389
**estimated_age, mean (SD)**		45.6 (23.5)
**gender, n (%)**	**FEMALE**	12915 (53.0)
	**MALE**	11391 (46.7)
	**UNKNOWN**	83 (0.3)
**race, n (%)**	**Native**	78 (0.3)
	**Asian**	2069 (8.5)
	**Black**	1079 (4.4)
	**Multirace**	494 (2.0)
	**NHPI**	108 (0.4)
	**Other Race**	3413 (14.0)
	**Unknown**	6535 (26.8)
	**White**	10613 (43.5)
**ethnicity, n (%)**	**Hispanic or Latino**	3815 (15.6)
	**Not Hispanic or Latino**	13869 (56.9)
	**Unknown**	6705 (27.5)
**# visits, mean (SD)**	missing = 3092	21.1 (51.8)

Supplemental Table 8: Hyperlipidemia UCDDP concepts and demographics

Top table shows the specific concepts utilized to identify HLD as the exposure in the UCDDP database, with breakdown by number of patients per concept. Due to deidentification, only a patient's birth year is known for age estimation. Recruitment age is utilized as the starting age for survival analysis, with HLD group as the age of HLD diagnosis, and unexposed group as the age of first EHR visit.

Term	# patients
Hyperlipidemia	702142
Mixed hyperlipidemia	169316

		Overall	No HLD	HLD	SMD
**n**		728578	364289	364289	
**gender, n (%)**	**FEMALE**	371050 (50.9)	186259 (51.1)	184791 (50.7)	0.037
	**MALE**	357255 (49.0)	177768 (48.8)	179487 (49.3)	
	**UNKNOWN**	273 (0.0)	262 (0.1)	11 (0.0)	
**race, n (%)**	**Native**	3278 (0.4)	1762 (0.5)	1516 (0.4)	0.113
	**Asian**	69432 (9.5)	32466 (8.9)	36966 (10.1)	
	**Black**	35072 (4.8)	16512 (4.5)	18560 (5.1)	
	**Multirace**	17486 (2.4)	7635 (2.1)	9851 (2.7)	
	**NHPI**	2972 (0.4)	1270 (0.3)	1702 (0.5)	
	**Other Race**	81646 (11.2)	44093 (12.1)	37553 (10.3)	
	**Unknown**	81062 (11.1)	44889 (12.3)	36173 (9.9)	
	**White**	437630 (60.1)	215662 (59.2)	221968 (60.9)	
**ethnicity, n (%)**	**H/L**	102163 (14.0)	53581 (14.7)	48582 (13.3)	0.126
	**Not H/L**	560067 (76.9)	271574 (74.5)	288493 (79.2)	
	**Unknown**	66348 (9.1)	39134 (10.7)	27214 (7.5)	
**estimated_age, mean (SD)**		69.7 (10.8)	69.6 (11.0)	69.8 (10.7)	0.012
**recruitment_age, mean (SD)**		63.9 (10.5)	63.4 (10.5)	64.3 (10.5)	0.087

Supplemental Table 9: Osteoporosis UCDDP concepts and demographics
Top table shows the specific concepts utilized to identify osteoporosis as the exposure in the UCDDP database with inclusion of children concepts, and breakdown by number of patients per concept. Due to deidentification, only a patient's birth year is known for age estimation. Recruitment age is utilized as the starting age for survival analysis, with osteoporosis group as the age of osteoporosis diagnosis, and unexposed group as the age of first EHR visit.

**Term**	# patients
Osteoporosis	145608
Senile osteoporosis	30611
Osteoporotic fracture	7772
Osteoporotic fracture of vertebra	3987
Localized osteoporosis - Lequesne	3126
Osteoporotic fracture of femur	2971
Idiopathic osteoporosis	1231
Disuse osteoporosis	309
Osteoporotic fracture of humerus	186
Osteoporotic fracture of hand	39

		**Overall**	**No osteo**	**osteo**	**SMD**
**n**		137880	68940	68940	
**gender, n (%)**	**FEMALE**	119637 (86.8)	60386 (87.6)	59251 (85.9)	0.049
	**MALE**	18241 (13.2)	8554 (12.4)	9687 (14.1)	
	**UNKNOWN**	2 (0.0)		2 (0.0)	
**race, n (%)**	**Native**	496 (0.4)	272 (0.4)	224 (0.3)	0.134
	**Asian**	15784 (11.4)	7364 (10.7)	8420 (12.2)	
	**Black**	4611 (3.3)	2546 (3.7)	2065 (3.0)	
	**Multirace**	3564 (2.6)	1737 (2.5)	1827 (2.7)	
	**NHPI**	419 (0.3)	198 (0.3)	221 (0.3)	
	**Other Race**	13032 (9.5)	7427 (10.8)	5605 (8.1)	
	**Unknown**	13670 (9.9)	7552 (11.0)	6118 (8.9)	
	**White**	86304 (62.6)	41844 (60.7)	44460 (64.5)	
**ethnicity, n (%)**	**H/L**	15530 (11.3)	8509 (12.3)	7021 (10.2)	0.133
	**Not H/L**	112474 (81.6)	54548 (79.1)	57926 (84.0)	
	**Unknown**	9876 (7.2)	5883 (8.5)	3993 (5.8)	
**estimated_age, mean (SD)**		74.8 (9.2)	75.2 (9.1)	74.5 (9.3)	-0.074
**recruitment_age, mean (SD)**		68.7 (8.9)	68.2 (8.7)	69.2 (9.1)	0.12
