# Supplementary material for: Leveraging electronic health records and knowledge networks for Alzheimer’s disease prediction and sex-specific biological insights
Source: Nat Aging. 2024 Feb 21;4(3):379–95. doi: 10.1038/s43587-024-00573-8 (PMC10950787; doi:10.1038/s43587-024-00573-8)
Supplement: Supplementary file 2 — Reporting Summary [file 43587_2024_573_MOESM2_ESM.pdf]

nature portfolio

Corresponding author(s): Marina Sirota

Last updated by author(s): Dec 20, 2023

Reporting Summary

Nature Portfolio wishes to improve the reproducibility of the work that we publish. This form provides structure for consistency and transparency in reporting. For further information on Nature Portfolio policies, see our Editorial Policies and the Editorial Policy Checklist.

Statistics

For all statistical analyses, confirm that the following items are present in the figure legend, table legend, main text, or Methods section.

n/a	Confirmed	
☐	☒	The exact sample size (*n*) for each experimental group/condition, given as a discrete number and unit of measurement
☒	☐	A statement on whether measurements were taken from distinct samples or whether the same sample was measured repeatedly
☐	☒	The statistical test(s) used AND whether they are one- or two-sided<br>*Only common tests should be described solely by name; describe more complex techniques in the Methods section.*
☐	☒	A description of all covariates tested
☐	☒	A description of any assumptions or corrections, such as tests of normality and adjustment for multiple comparisons
☐	☒	A full description of the statistical parameters including central tendency (e.g. means) or other basic estimates (e.g. regression coefficient) AND variation (e.g. standard deviation) or associated estimates of uncertainty (e.g. confidence intervals)
☐	☒	For null hypothesis testing, the test statistic (e.g. *F*, *t*, *r*) with confidence intervals, effect sizes, degrees of freedom and *P* value noted<br>*Give P values as exact values whenever suitable.*
☒	☐	For Bayesian analysis, information on the choice of priors and Markov chain Monte Carlo settings
☒	☐	For hierarchical and complex designs, identification of the appropriate level for tests and full reporting of outcomes
☐	☒	Estimates of effect sizes (e.g. Cohen's *d*, Pearson's *r*), indicating how they were calculated

Our web collection on statistics for biologists contains articles on many of the points above.

Software and code

Policy information about availability of computer code

Data collection	No new data was collected for this study. No software was used for data collection.
Data analysis	Code for prediction models can be found at github.com/al1563/ADprediction_code.<br>Relevant packages:<br>Python<br>- joblib version 1.1.0<br>- scikit-learn version 0.23.2<br>- scipy version >= 1.2.0<br>- lifelines version 0.27.4<br>- tableone version 0.7.12<br>R<br>- coloc version 5.1.0

For manuscripts utilizing custom algorithms or software that are central to the research but not yet described in published literature, software must be made available to editors and reviewers. We strongly encourage code deposition in a community repository (e.g. GitHub). See the Nature Portfolio guidelines for submitting code & software for further information.

Data

Policy information about availability of data

All manuscripts must include a data availability statement. This statement should provide the following information, where applicable:

- Accession codes, unique identifiers, or web links for publicly available datasets
- A description of any restrictions on data availability
- For clinical datasets or third party data, please ensure that the statement adheres to our policy

EHR data is obtained from the UCSF or UC-wide de-identified data warehouse. These datasets are restricted due to the sensitive nature of the data, but affiliated individuals can request access, or otherwise set up an official collaboration with an affiliated lab. EHR concepts to identify cohorts and controls are described in Methods and in Supplemental Tables 1 and 2. Further information about specific concepts utilized in models can be found in Supplemental Data.

Phecodes can be downloaded at phewascatalog.org/phecodes_icd10 or phewascatalog.org/phecodes, and mappings between ICD-10 codes and SNOMED can be accessed at www.nlm.nih.gov/healthit/snomedct/us_edition.html. Open Targets Genetics can be accessed at genetics.opentargets.org, and molecular QTL data can be queried by their API to identify the source publication. Data for UK Biobank phenotype GWAS can be found at www.nealelab.is/uk-biobank/ (sex-stratified heel bone mineral density: phenotype code 3148_irnt), and cis-eQTL data can be downloaded from www.eqtlgen.org/. Demographics and covariates can be found in the original publications. The SPOKE knowledge network can be accessed at spoke.rbvi.ucsf.edu/, and more details about the network can be found in Morris et al. and mappings to EHR concepts can be found in Nelson et al.

Human research participants

Policy information about studies involving human research participants and Sex and Gender in Research.

Reporting on sex and gender	This study investigated sex as a biological variable, and identified individuals into sex category based on available EHR data.
Population characteristics	Machine learning models are trained on individuals seen at UCSF, and clinical validation done on individuals seen at a University of California health center represented in the UC-wide database. Demographic information including race and ethnicity are reported in relevant Tables or Supplement Tables. Further genetic validation was performed on prior published cohorts (which often includes individuals of European ancestry), with relevant studies referenced in the publication.<br><br>Within the UCSF EHR, there are 5,582,007 patients. With filtering, 251,294 patients were utilized for analysis.140,016 (55.7%) are female identifying, with birth year on average in year 1945 (with average age 62.5 in the -7 year model and 69.5 in the -1 day model). In terms of race and ethnicity, 158,232 (63%) are white-identifying, 17,173 (6.8%) are black-identifying, 32,578 (13%) are asian-identifying, and 15,089 (6%) are categorized as latinx (note that at UCSF, race & ethnicity is a single variable derived from an algorithm developed by the UCSF Data Equity Taskforce to codify aggregated sociopolitical categorizations based on EHR self-reported identifiers).<br><br>Within the UC-wide data, for hyperlipidemia there are 364,289 patients. 184791 (50.7%) are female. In terms of race and ethnicity, 36,966 (10.1) are asian-identifying, 18,560 (5.1%) are black-identifying, 221,968 (60.9%) are white-identifying, and 48,582 (13.3%) are categorized as hispanic/latino. The estimated age is 64.3 (10.5 sd) at diagnosis (or study recruitment). Controls matched on these characteristics (more in Supplementary Table 8).<br>For osteoporosis, there are 68,940 patients, 59,251 (85.9%) female. 8,420 (12.2%) are asian-identifying, 2,065 (3.0%) are black-identifying, 44,460 (64.5%) are white-identifying, and 7,021 (10.2%) are categorized as hispanic/latino. The estimated age is 69.2 (9.1 sd) at diagnosis / recruitment.
Recruitment	No recruitment was performed.
Ethics oversight	This study was approved by the Institutional Review Board of University of California San Francisco (IRB #20-32422).

Note that full information on the approval of the study protocol must also be provided in the manuscript.

Field-specific reporting

Please select the one below that is the best fit for your research. If you are not sure, read the appropriate sections before making your selection.

☒ Life sciences ☐ Behavioural & social sciences ☐ Ecological, evolutionary & environmental sciences

For a reference copy of the document with all sections, see nature.com/documents/nr-reporting-summary-flat.pdf

Life sciences study design

All studies must disclose on these points even when the disclosure is negative.

Sample size	No sample size calculation was performed prior to study. Sample size was determined by the number of patients with Alzheimer's Disease in the UCSF database (2,996 AD and 823,671 controls) and sufficient temporal and informational capture in the EHR (over 7 years with at least a

	clinical visit or record, 749 AD patients and 250,545 controls). Training for matched cohort models was performed on a 1:8 propensity-score matched cohort to allow for both sufficient patient balancing and represent the rate of AD in the population.
Data exclusions	Controls were excluded if diagnosed with dementia (Supplemental Table 1) to increase confidence in capturing controls without AD or prodromal AD. Both AD and control patients were filtered to include patients at least 55 years of age at index time to capture sufficient number of patients and data before AD onset (note that AD diagnosis may be given past index time).
Replication	Not applicable. While this study did not replicate, validation was performed in the University of California EHR system and with prior published genetic/molecular datasets.
Randomization	Not applicable because this study did not acquire new data and therefore randomization is not possible in observational datasets like the EHR. Pseudo-randomization was performed with propensity score matching, an approach in causal inference to match by probability of group membership, to enable identification of matching case and control groups and mimic randomization. Quality of matching can be assessed with standardized mean difference of relevant covariates and is shown in relevant patient characteristic tables.
Blinding	Not applicable because this study did not acquire new data and utilized data acquired from health care use and prior publications.

Reporting for specific materials, systems and methods

We require information from authors about some types of materials, experimental systems and methods used in many studies. Here, indicate whether each material, system or method listed is relevant to your study. If you are not sure if a list item applies to your research, read the appropriate section before selecting a response.

Materials & experimental systems

n/a	Involved in the study
☒	☐ Antibodies
☒	☐ Eukaryotic cell lines
☒	☐ Palaeontology and archaeology
☒	☐ Animals and other organisms
☒	☐ Clinical data
☒	☐ Dual use research of concern

Methods

n/a	Involved in the study
☒	☐ ChIP-seq
☒	☐ Flow cytometry
☒	☐ MRI-based neuroimaging
